# An in vitro approach reveals molecular mechanisms underlying endocrine disruptor-induced epimutagenesis

**DOI:** 10.7554/eLife.93975

**Published:** 2024-10-03

**Authors:** Jake D Lehle, Yu-Huey Lin, Amanda Gomez, Laura Chavez, John R McCarrey

**Affiliations:** 1 Department of Neuroscience, Developmental and Regenerative Biology, The University of Texas at San Antonio San Antonio United States; ETH Zurich Switzerland; The Lundquist Institute United States

**Keywords:** endocrine disrupting chemicals, epigenetics, DNA methylation, epigenetic reprogramming, transgenerational epigenetic inheritance, Mouse

## Abstract

Endocrine disrupting chemicals (EDCs) such as bisphenol S (BPS) are xenobiotic compounds that can disrupt endocrine signaling due to steric similarities to endogenous hormones. EDCs have been shown to induce disruptions in normal epigenetic programming (epimutations) and differentially expressed genes (DEGs) that predispose disease states. Most interestingly, the prevalence of epimutations following exposure to many EDCs persists over multiple generations. Many studies have described direct and prolonged effects of EDC exposure in animal models, but many questions remain about molecular mechanisms by which EDC-induced epimutations are introduced or subsequently propagated, whether there are cell type-specific susceptibilities to the same EDC, and whether this correlates with differential expression of relevant hormone receptors. We exposed cultured pluripotent (iPS), somatic (Sertoli and granulosa), and primordial germ cell-like (PGCLC) cells to BPS and found that differential incidences of BPS-induced epimutations and DEGs correlated with differential expression of relevant hormone receptors inducing epimutations near relevant hormone response elements in somatic and pluripotent, but not germ cell types. Most interestingly, we found that when iPS cells were exposed to BPS and then induced to differentiate into PGCLCs, the prevalence of epimutations and DEGs was largely retained, however, >90% of the specific epimutations and DEGs were replaced by novel epimutations and DEGs. These results suggest a unique mechanism by which an EDC-induced epimutated state may be propagated transgenerationally.

## Introduction

It has now been more than a half-century since Roy Hertz in 1958 first proposed the notion that certain chemicals, particularly those used in livestock feed at the time, could contaminate food sources and bioaccumulate in humans mimicking the activity of hormones (Gassner et al., 1958). As the number of potential chemicals that could exert these effects has grown, so too has the interest in this area to the point that this is now a major topic of active research. The dangers of many endocrine disrupting chemicals (EDCs) studied to date have largely been investigated utilizing animal models. A primary example is the multi-phased Toxicant Exposures and Responses by Genomic and Epigenomic Regulators of Transcription (TaRGET) program established to determine the contribution of environmental exposures to disease pathogenesis as a function of epigenome perturbation in mouse models (Wang et al., 2018). While the use of animal models has been quite informative for determining the various systemic or organ/tissue-specific disease-related effects associated with exposure to different EDCs, the molecular mechanisms underlying these phenomena remain poorly understood. Thus, questions persist regarding (i) the mechanisms by which exposure of cells to EDCs results in the initial formation of epimutations defined here as any change in epigenomic programming, (ii) potential cell-type specific differential susceptibility to epimutagenesis induced by different EDCs, (iii) the potential involvement of relevant hormone receptors and/or genomic hormone response elements in EDC-induced disruption of the epigenome, and (iv) the ability of an EDC-induced epimutated state to persist inter- or transgenerationally despite generational epigenetic reprogramming (Diaz-Castillo et al., 2019).

Questions about molecular mechanisms responsible for EDC-exposure based disruption of normal epigenetic programming have been difficult to interpret in whole animal models due, in part, to the inherent complexity of the intact animal including potential interactions among multiple different organs, tissues, and cell types at the paracrine, metabolic and/or systemic levels. To circumvent this challenge, we opted to expose homogeneous populations of specific cell types in culture to doses of the EDC, Bisphenol S (BPS), that are below the maximum safe limit previously set by the EPA for exposure of humans to the similar estrogen mimetic, Bisphenol A (BPA). In this way, we hoped to learn more about the manner in which direct exposure to a specific EDC induces epimutations and differential gene expression in different individual cell types including somatic cells, pluripotent cells, and germ cells. This approach was designed to distinguish the direct effects of an EDC exposure in vitro from indirect effects that may accrue as the result of an EDC exposure on one cell type inducing a secondary effect on a neighboring or related cell type in vivo.

Previous in vitro studies have demonstrated direct susceptibility of cultured cell types to EDC-induced epimutagenesis, however, those studies were focused on immortalized cancer cell lines that do not necessarily model key cell types involved in normal initial incursion or subsequent intragenerational propagation or inter- or transgenerational transmission of environmentally induced epimutations in vivo (Deb et al., 2016; Goodman et al., 2014; Huang et al., 2019; Karaman and Ozden, 2019; Senyildiz et al., 2017). We chose to examine direct exposure of three key types of cells relevant to environmental exposures and subsequent propagation and transmission of induced epimutations in vivo – somatic cell types known to be responsive to endocrine signaling (Sertoli cells and granulosa cells), pluripotent cells mimicking the preimplantation embryo (iPSCs), and germline cells (PGCLCs).

While there have been reports that have clearly indicated the ability of EDCs to disrupt classical endocrine signaling (Henley and Korach, 2010; Kelce et al., 1995; Swedenborg et al., 2009; vom Saal and Hughes, 2005; You et al., 1998), there exist other reports indicating EDCs can also disrupt non-classical endocrine signaling via nuclear receptors (Ozgyin et al., 2015; Myers et al., 2011), G protein-coupled receptors (Thomas and Dong, 2006), and calcium channel signaling (Brenker et al., 2018). This has further complicated our understanding of the mechanism(s) by which EDC exposure initially induces epimutations, and has particularly confounded insight into the susceptibility of germ cells to EDC-induced epimutagenesis given that germ cells are reported to lack expression of classical endocrine receptors (Meccariello et al., 2014). This is relevant to the effort to understand mechanisms underlying transgenerational epigenetic inheritance of EDC-induced epimutations (Anway et al., 2006; Guerrero-Bosagna et al., 2012; Nilsson et al., 2008) which clearly implicates transmission via the germ line.

The concept of germline transmission of EDC-induced epimutations is further confounded by the known epigenetic reprogramming events that occur in the preimplantation embryo and developing fetal and neonatal germ line. Thus, regardless of how EDC-induced epimutations become initially manifest in the germ line, it is not clear how they subsequently persist and are transmitted inter- or transgenerationally given the large portion of the epigenome that undergoes genome-wide erasure and resetting of epigenetic programming during each generation (Cantone and Fisher, 2013; Lee et al., 2014; Santos et al., 2002; Sanz et al., 2010). In vitro cell culture systems afford the opportunity to focus on individual cell types independently, including somatic cells which are likely initially exposed to most environmental disruptive influences, pluripotent cells which represent the preimplantation embryo in which the first major reprogramming event occurs during normal development, and early germline cells where the second major reprogramming event is initiated in primordial germ cells (PGCs). Importantly, in addition to the potential to study each of these cell types independently, it is possible to induce transitions in cell fate in vitro that model those that occur during normal development, thereby recapitulating normal reprogramming events in a way that can facilitate high resolution studies of the fate of EDC-induced epimutations once they have been induced in any of these cell types.

Here we describe our study of the relative susceptibility to induction of epimutations by direct exposure of four different cell types maintained in culture – somatic Sertoli and granulosa cells, pluripotent iPSCs, and germline PGCLCs – to BPS, followed by our analysis of the fate of epimutations initially induced in mouse iPSCs that are then induced to transition into PGCLCs. We found that there are cell-type specific differences in susceptibility to epimutagenesis and associated dysregulation of gene expression patterns following exposure of these different cell types to a similar dose of BPS below the maximum safe limit established by the EPA for exposure of human cells to BPA (Ribeiro et al., 2019). We further found that BPS induction of epimutations in both pluripotent and somatic cell types that express relevant estrogen receptors (ERs), as well as in germ cells which do not express ERs, suggests disruption of both canonical and non-canonical endocrine signaling. Most interestingly, we found that when iPSCs exposed to BPS were then induced to undergo a major transition in cell fate and related epigenetic reprogramming to form PGCLCs, a similar prevalence of epimutations and DEGs was retained, but, surprisingly, very few (<10%) specific epimutations or DEGs were conserved during this process. This suggests that the initial EDC exposure induces disruption of the chromatin landscape that subsequent epigenetic reprogramming is unable to fully restore. Thus, reprogramming during a major cell fate transition appears to correct many of the initially induced epimutations, but also appears to induce many de novo epimutations, and this imbalance may persist across multiple generations which may contribute to continued transgenerational epigenetic inheritance of EDC-induced phenotypes during succeeding generations.

## Results

### Dose-dependent epimutagenesis response to BPS exposure in iPSCs

**Figure 1.**
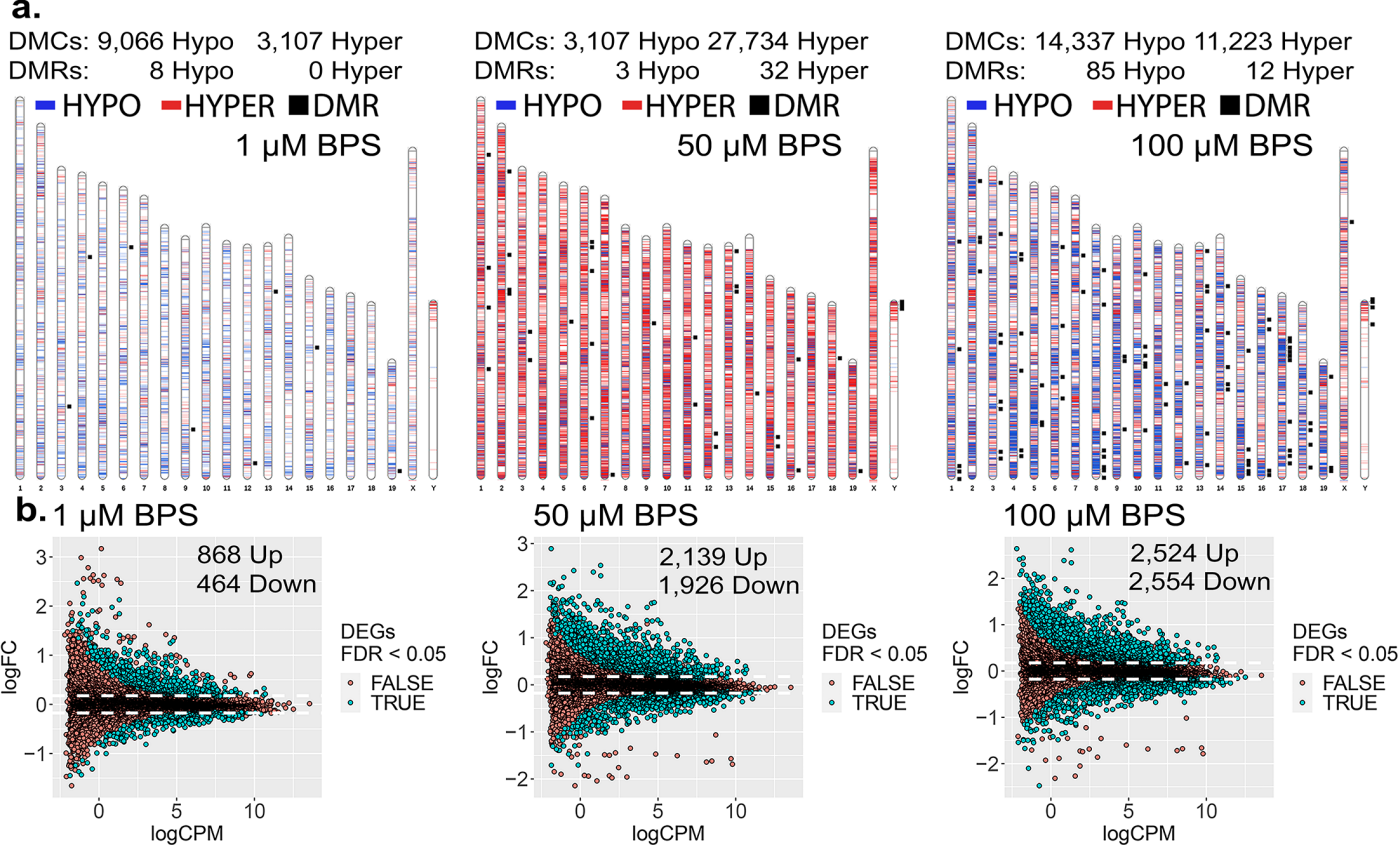
Dose-dependent impact of epimutagenesis measured in iPSCs exposed to 1, 50, and 100 μM BPS. (**a**) Ideogram plots displaying chromosomal distribution of genome-wide changes in DNA methylation caused by BPS exposure. (**b**) Mean difference (MD) plots of changes in gene expression following exposure to increasing doses of BPS. Exposure to increasing doses of BPS induced higher, although plateauing numbers of DMCs, DMRs, and DEGs. Blue horizontal lines = hypomethylated DMCs, red horizontal lines = hypermethylated DMCs, black squares = DMRs.

To initially assess the extent to which epimutations and dysregulated gene expression can be induced in cells maintained in culture, we exposed mouse iPSCs to three doses of BPS (1, 50, and 100 μM) and measured the impact on the epigenome and transcriptome using the Illumina Infinium Mouse Methylation BeadChip Array and RNA-seq, respectively (Figure 1). All three doses of BPS induced individual differentially methylated CpGs (DMCs) as well as differentially methylated regions (DMRs) (Figure 1a), and differentially expressed genes (DEGs) (Figure 1b) when compared to control mouse iPSCs treated with vehicle (EtOH) only. The extent of this exposure-specific epimutagenesis was correlated with the dose of BPS used. Information regarding the overlap of DMCs/DMRs/DEGs identified for each dose of BPS is shown in Figure 1—figure supplement 1. We observed BPS-induced DMCs within both promoter and gene body regions of a portion of DEGs (17-38%) (Table 1). Importantly, in many cases, we observed a correlation between differential expression of a gene and the presence of DMCs in the promoter region of that gene (Figure 1—figure supplement 2a). These results provided an initial proof of concept that exposure of cells maintained in vitro to an EDC such as BPS can disrupt the epigenome and transcriptome in a dose-dependent manner. Interestingly, exposure to 1 µM BPS induced very few DMRs, but did induce widespread DMCs and DEGs. Because 1 μM BPS is below the FDA’s suggested safe environmental level established for exposure of humans or intact animals to BPA (Ribeiro et al., 2019), and was sufficient to induce DMCs and dysregulated gene expression on all chromosomes in our cultured iPSCs, we utilized this dose and focused solely on DMCs when assessing epimutations in all subsequent experiments (Table 1).

**Figure 1—figure supplement 1.**
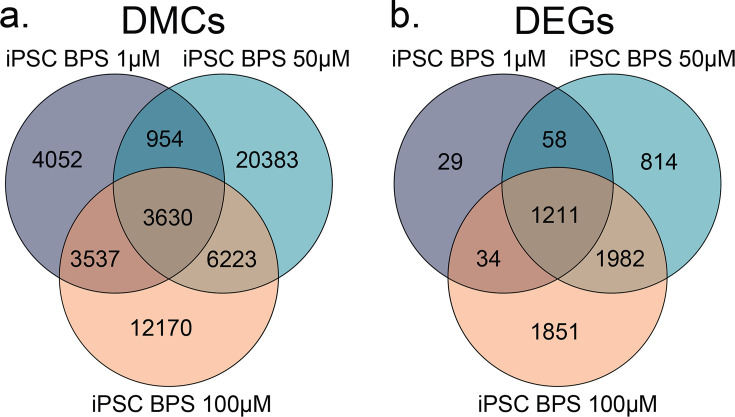
Overlapping DMCs and DEGs found among dose-dependent responses to BPS exposure. Venn diagrams of overlapping (**a**) DMCs and (**b**) DEGs identified when comparing iPSCs exposed to increasing doses of BPS (1, 50, and 100 μM). We detected an average of 51.25% overlap among DMCs and an average of 80.45% overlap among DEGs within each respective cell type across the different doses of BPS.

**Figure 1—figure supplement 2.**
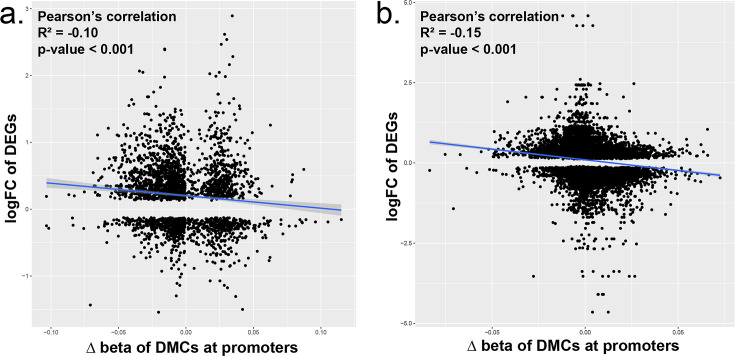
Relationship between DMCs at promoters and DEGs. We compared the correlation between the differential expression of genes and the presence of DMCs in the promoter region of that gene in (**a**) iPSCs exposed to increasing doses of BPS (1, 50, and 100 μM) or (**b**) Sertoli, Granulosa, iPSCs, and PGCLCs. We found that there was a significant negative correlation indicating in our data that promoters that had a loss of DNA methylation tended to also have higher upregulation of gene expression and vice versa when observing hypermethylation and downregulation of gene expression.

**Figure 1—figure supplement 3.**
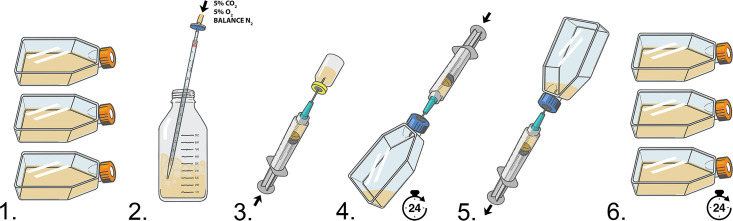
Chemical exposure experimental workflow. (**1**) Cells are passaged into T-25 flasks with filter caps. (**2**) Mixed blood gas (carbon dioxide 5%, oxygen 5%, and balance nitrogen) is filtered and bubbled into media to prepare media to be mixed with diluted chemical treatment and added to air-tight cell culture flasks. (**3**) Media is transferred into glass vials and diluted chemical is added. (**4**) T-25 filter caps are replaced with air-tight caps with septums and cell media containing chemicals is added via syringe and left for 24 hr. (**5**) After 24 hr, chemical-containing media is removed and cells are washed with buffer. (**6**) T-25 air-tight septum caps are replaced with filter caps and cells are cultured for an additional 24 hr ‘chase’ period.

**Figure 1—figure supplement 4.**
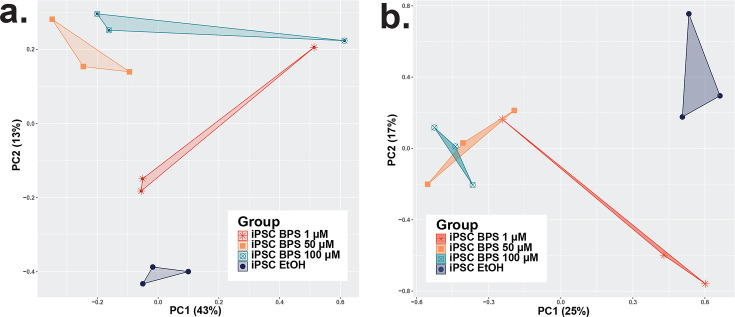
Consistency among iPSC replicates and variation between RNA-seq and DNA methylation Infinium Beadchip array experimental and control groups. Plots displaying principle component analyses of data from the BPS dose determination experiments showing dose-dependent susceptibility to BPS in iPSCs utilizing data from (**a**) DNA methylation data from Infinium Beadchip array analysis and (**b**) gene expression data from bulk RNA-seq analysis. The dimensional reduction of the variation in plots demonstrates a partially additive dose-dependent relationship between the concentration of BPS added to the media and an increasing distinction between the control and treated samples, although this relationship plateaued at higher doses of BPS.

**Figure 1—figure supplement 5.**
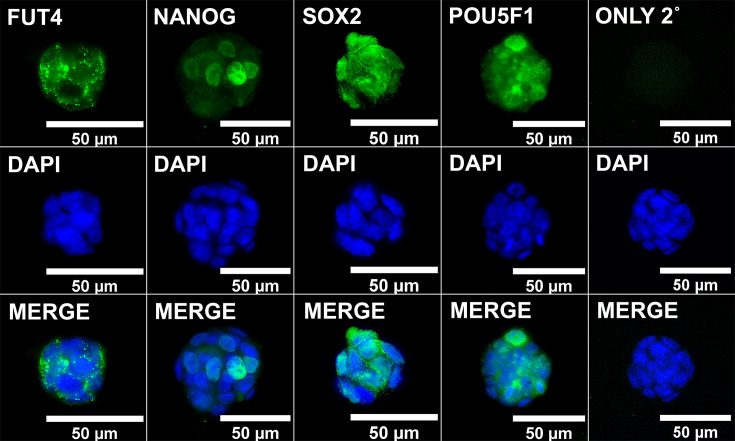
ICC validation of MF5-9-1 iPSCs. iPSCs from reprogrammed MEFs were validated for immunolabeling with known pluripotency markers along with negative control results when labeling was conducted with the secondary antibody only (ONLY 2°).

**Table 1.** DEGs containing DMCs observed in iPSC exposed to increasing doses of BPS.

DEGs containing DMCs	iPSC 1 μ**M**	iPSC 50 μ**M**	iPSC 100 μ**M**
Promoter	264 (19.82%)	693 (17.04%)	1136 (22.37%)
Gene body	436 (32.73%)	1541 (37.91%)	1934 (38.08%)

### Cell-type specific susceptibility to induction of epimutations following BPS exposure

**Figure 2.**
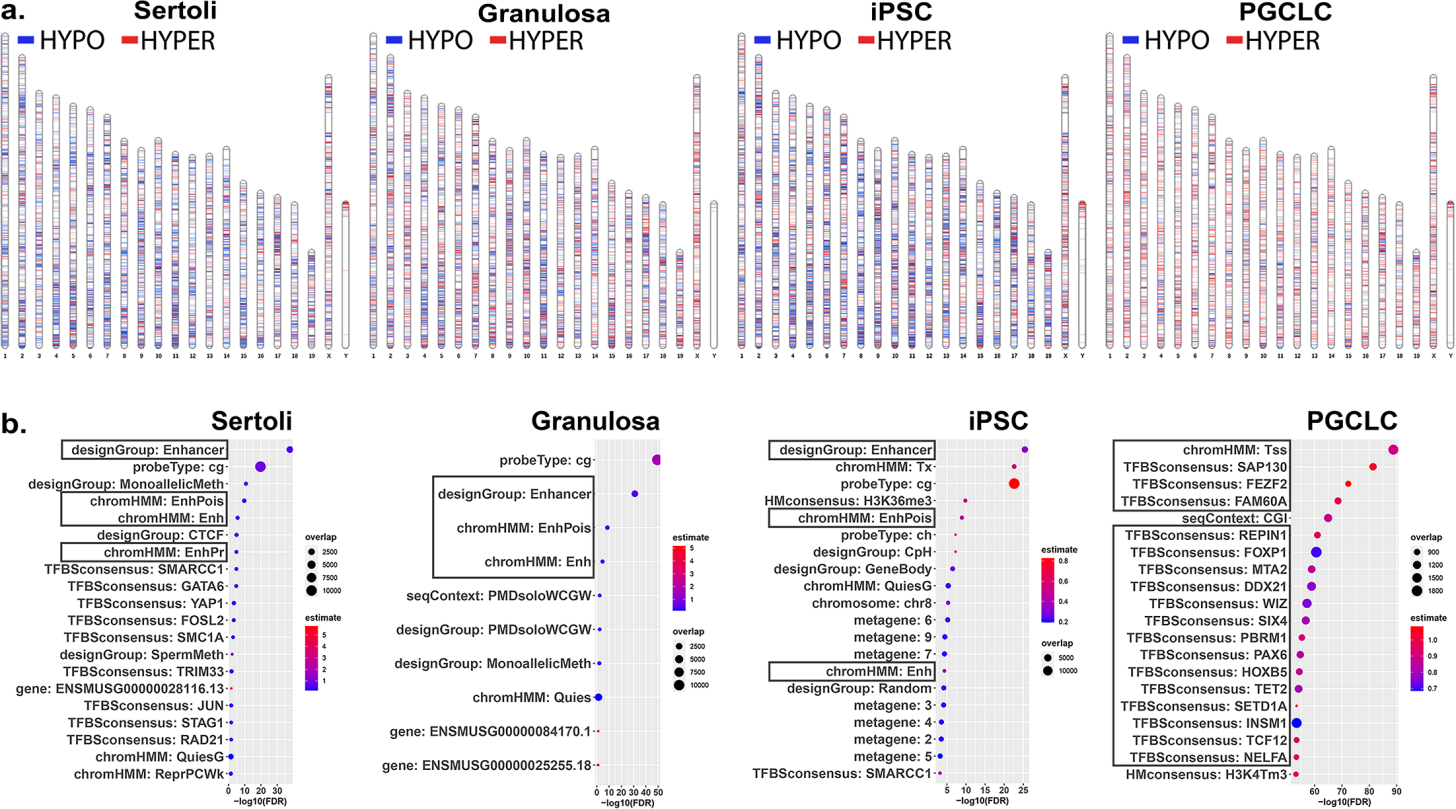
Chromosomal distributions and annotations of BPS-induced epimutations in pluripotent, somatic, and germ cell types. (**a**) Ideograms illustrating chromosomal locations of DMCs induced by exposure of each cell type to 1 μM BPS. Blue horizontal lines = hypomethylated DMCs, red horizontal lines = hypermethylated DMCs. (**b**) Enrichment plots indicating feature annotations in genomic regions displaying prevalent BPS-induced epimutations in each cell type. Dot size = number of overlapping DMCs with specific annotation, dot color = enrichment score reflecting the relative degree to which epimutations occurring in a specific annotated class are overrepresented.

We next sought to determine if different key cell types – somatic, pluripotent or germ – are differentially susceptible to induction of epimutations in response to direct exposure of each to a similar dose of BPS. Thus, we exposed pluripotent (iPSCs), somatic (Sertoli and granulosa cells), and germ (PGCLCs) cell types to 1 μM BPS and measured changes in DNA methylation patterns. We identified exposure-specific DMCs in each exposed cell type relative to its corresponding control (exposed to carrier only; Figure 2a). We observed overall differences among the different cell types in total numbers of DMCs, with iPSCs showing the highest number of DMCs, followed by Sertoli cells and granulosa cells, and then PGCLCs, respectively (Table 2).

**Figure 2—figure supplement 1.**
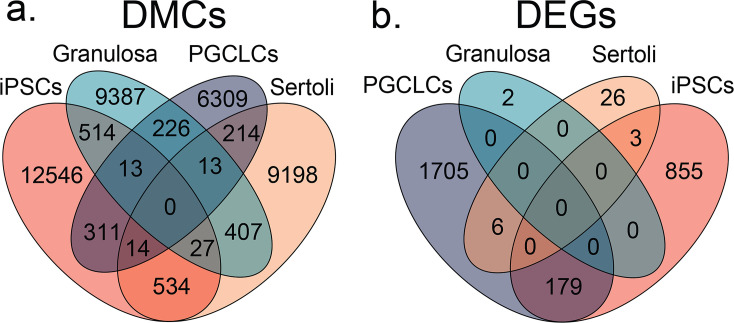
Overlapping DMCs and DEGs found among cell-type specific responses to BPS exposure. Venn diagrams of overlapping (**a**) DMCs and (**b**) DEGs identified when comparing iPSCs, granulosa cells, Sertoli cells, and PGCLCs exposed to the established minimum dose of 1 μM of BPS. We only detected an average of 11.05% among DMCs and 13.26% among DEGs between different cell types.

**Figure 2—figure supplement 2.**
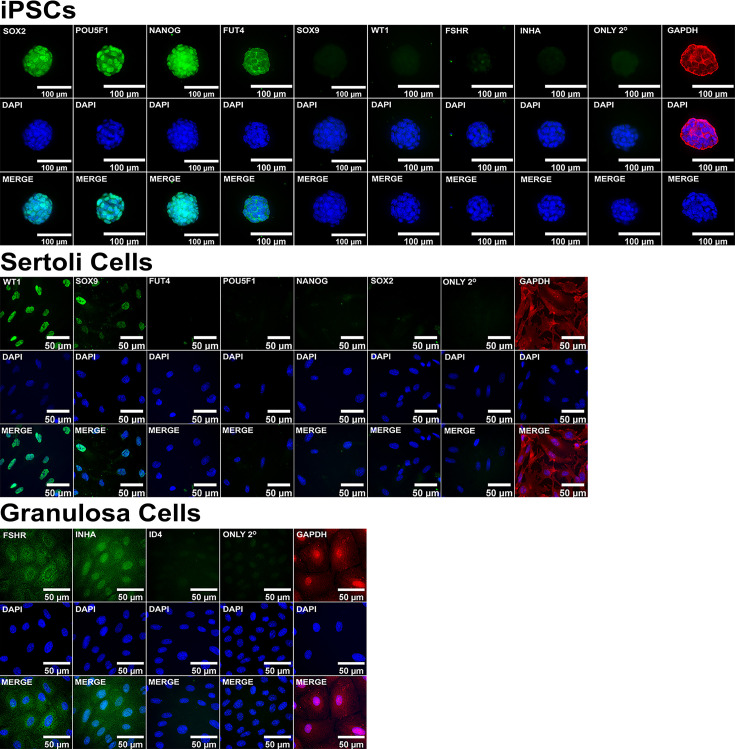
ICC control staining of cell type-specific markers. Validation of immunolabeling for known pluripotent and somatic cell type markers in iPSCs, Sertoli Cells, and Granulosa cells along with negative secondary antibody only (ONLY 2°) and positive (GAPDH) controls.

**Figure 2—figure supplement 3.**
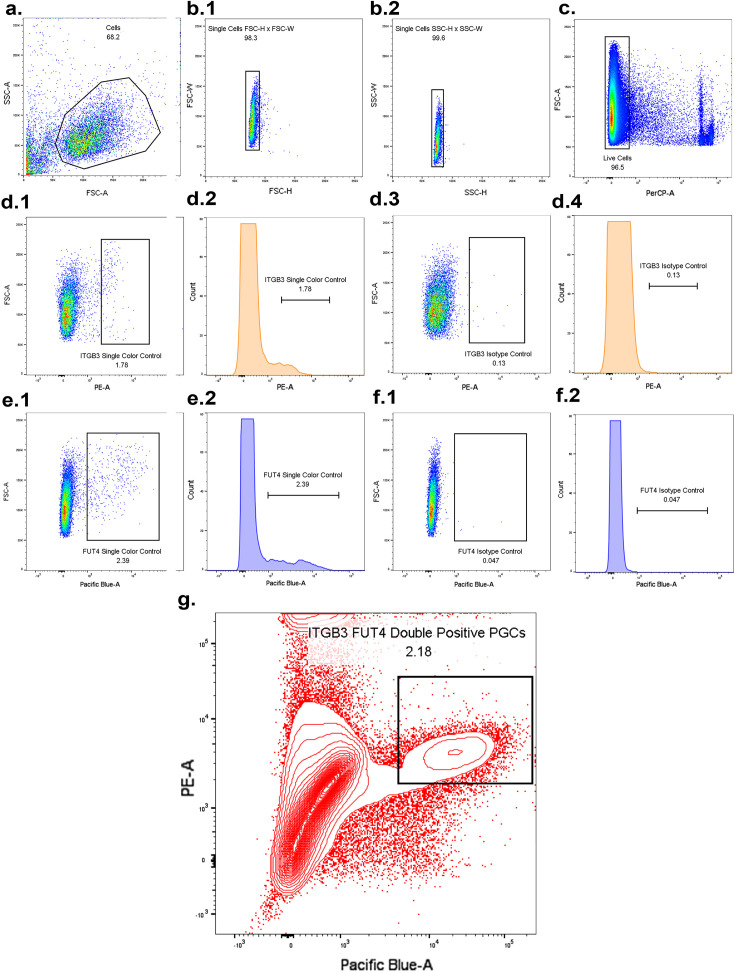
FACS sorting for ITGB3/FUT4 enriched primordial germ-cell like cells. (**a**) Gating for cells. (**b**) (1–2) Gating for singlet cells. (**c**) Gating for live cells. (**d**) (1–2) Single color control ITGB3-positive cells. (**d**) (3–4) IgG isotype control. (**e**) (1–2) Single color control FUT4-positive cells. (**f**) (3–4) IgG isotype control. (**g**) Sorting for PGCLC-enriched ITGB3/FUT4 double positive population (2.18% of total cells).

**Figure 2—figure supplement 4.**
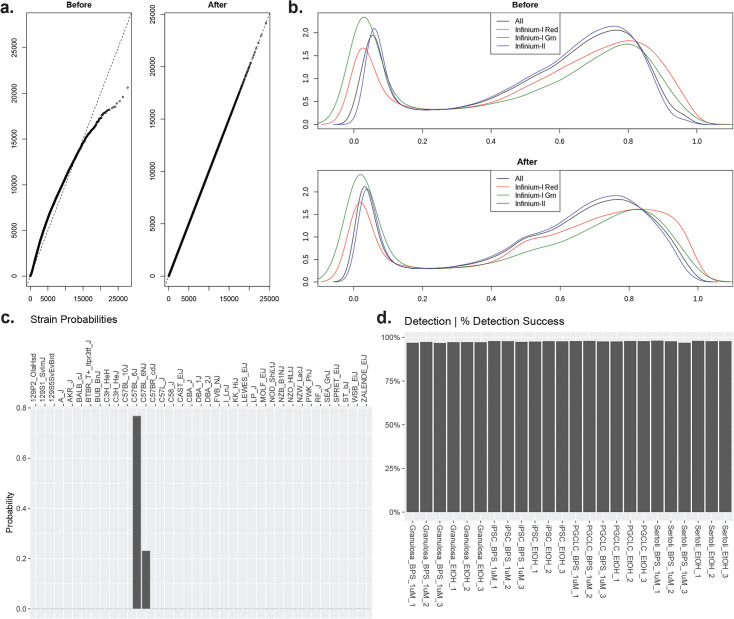
Quality control for Infinium Mouse Methylation BeadChip Array data. (**a**) Non-linear correction of dye bias removal from sample data. (**b**) Background signal subtraction from samples to limit noise. (**c**) Prediction of correct C57B6 mouse strain from samples included in the study based on built-in controls on Infinium Mouse Methylation BeadChip Array. (**d**) Average CpG probe detection success of 97.58% across all samples indicating efficient bisulfite conversion of all samples.

**Table 2.** Treatment-specific differentially methylated sites (DMCs) (treated vs. control).

DMCs	Sertoli	Granulosa	iPSCs	PGCLCs
Hypomethylated*	7385	6444	9651	2315
Hypermethylated^†^	3022	4143	4308	4785
Total	10,407	10,587	13,959	7100

* A CpG site that was predominantly methylated in the control samples but unmethylated in the exposed samples.

† A CpG site that was predominantly unmethylated in the control samples but methylated in the exposed samples.

Interestingly, among the exposure-specific DMCs identified in each cell type, we observed predominantly hypomethylated DMCs in the somatic and pluripotent cell types, but predominantly hypermethylated DMCs in PGCLCs (Table 2). These findings confirm that there are both quantitative differences among cell types in overall susceptibility to epimutagenesis, and qualitative cell-type specific differences in the prevalence of hypo- versus hypermethylated DMCs following exposure to BPS. The latter likely reflects the fact that the epigenome in PGCs is naturally more hypomethylated than that in pluripotent or somatic cell types (Hajkova, 2011), enhancing the likelihood that most changes in DNA methylation induced in PGCLCs will necessarily involve hypermethylation.

### Annotation of BPS-induced epimutations

As we only found an average of 11.05% direct overlap in DMCs between two or more cell types and no overlapping DMCs shared between all cell types (Figure 2—figure supplement 1), we next analyzed annotations associated with genomic sites of BPS exposure-specific DMCs in each cell type. We mined annotations associated with genomic regions included on the Infinium array and found that a substantial group of exposure-specific DMCs was associated with enhancer regions in the two somatic (Sertoli and granulosa) and one pluripotent (iPS) cell types exposed to BPS, while in PGCLCs BPS-induced DMCs were more prevalent at promoter regions containing transcription start sites (TSSs) and transcription factor binding sites (Figure 2b), indicative of yet another qualitative cell-type specific difference in induction of epimutations by the same dose of BPS.

### Relationship between susceptibility to BPS-induced epimutagenesis and expression of relevant hormone receptors

**Figure 3.**
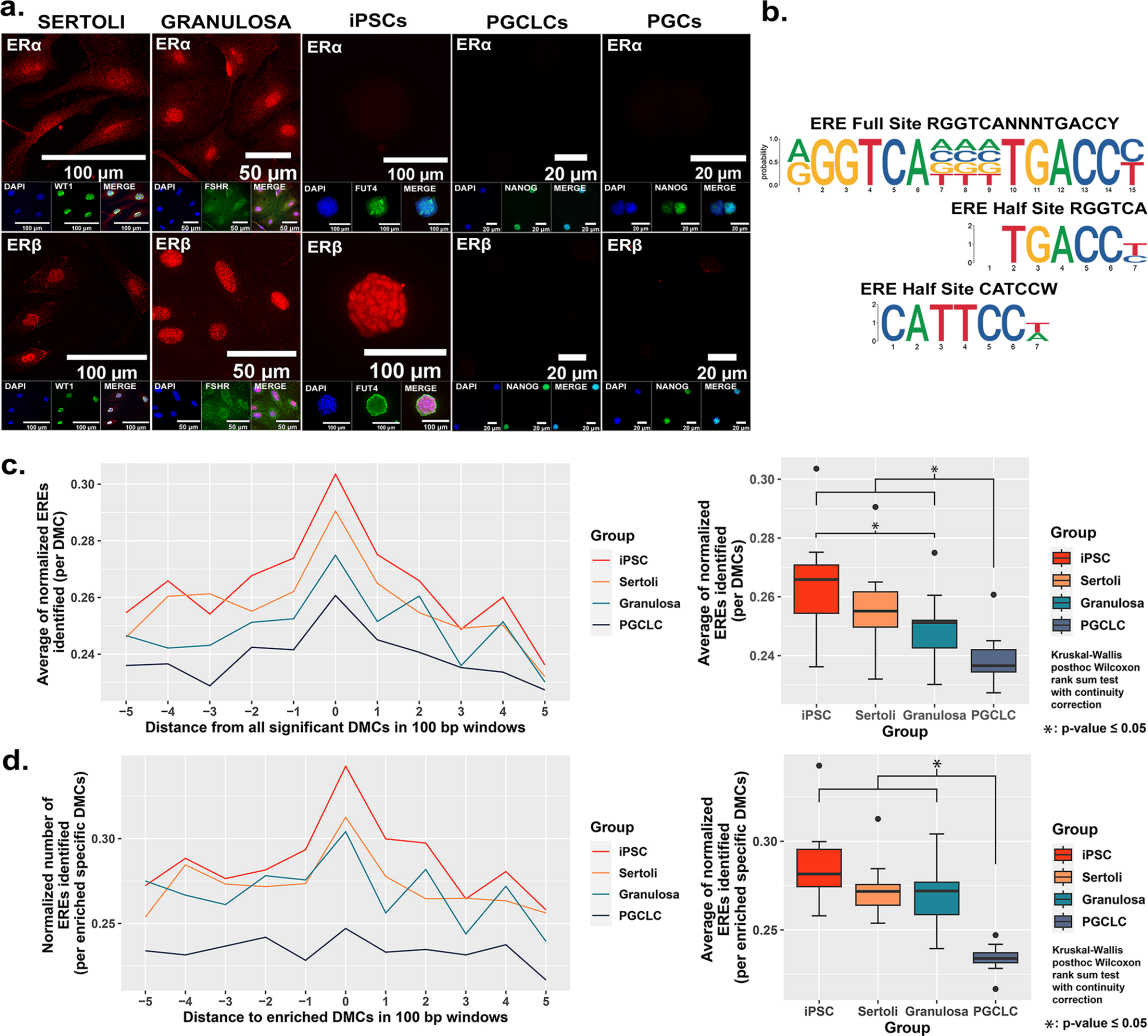
Correlation between cell-type specific expression of estrogen receptors and density of genomic EREs associated with BPS-induced epimutations. (**a**) Assessment of expression of ERα and ERβ by cell types co-stained for known cell-type specific markers. Somatic cell types express both receptors, pluripotent cells express ERβ but not ERα, and germ cells do not express either estrogen receptor. (**b**) Motif plots displaying the full ERE consensus sequence and the more biologically relevant ERE half-site motifs found to be enriched from ERα ChIP-seq. (**c,d**) Normalized density plots and box plots displaying the frequency of ERE half-sites identified (**c**) within 500 bp of all BPS-induced DMCs genome-wide, or (**d**) within 500 bp of the most enriched categories of BPS-induced DMCs in each cell type (=enhancer regions for somatic and pluripotent cell types and promoter regions in the germ cell type).

As EDCs are thought to induce epimutations via disruption of classical hormonal signaling (Henley and Korach, 2010; Kelce et al., 1995; Swedenborg et al., 2009; vom Saal and Hughes, 2005; You et al., 1998), we next sought to determine if the differential extent of BPS induction of epimutations we observed in different cell types was associated with differential expression of relevant hormone receptors in each. We performed immunocytochemistry (ICC) to detect the presence of the relevant estrogen receptors – ERα and ERβ, while co-staining for cell-type specific markers (WT1 [Sertoli cell marker], FSHR [granulosa cell marker], FUT4 [iPSC marker], and NANOG [PGCLC or endogenous PGC marker]) to confirm the identity of the four cultured cell types examined in this study (Sertoli cells, granulosa cells, iPSCs and PGCLCs), as well as endogenous mouse PGCs (Figure 3a). Both somatic cell types (Sertoli and granulosa) showed positive immunolabeling for both ERα and ERβ, whereas the pluripotent cells showed positive immunolabeling for ERβ, but negative immunolabeling for ERα, and the PGCLCs and endogenous PGCs showed negative immunolabeling for both ERα and ERβ. Importantly, the latter result is consistent with previous reports of lack of expression of either ERα or ERβ at the protein level in endogenous PGCs (Meccariello et al., 2014).

**Figure 3—figure supplement 1.**
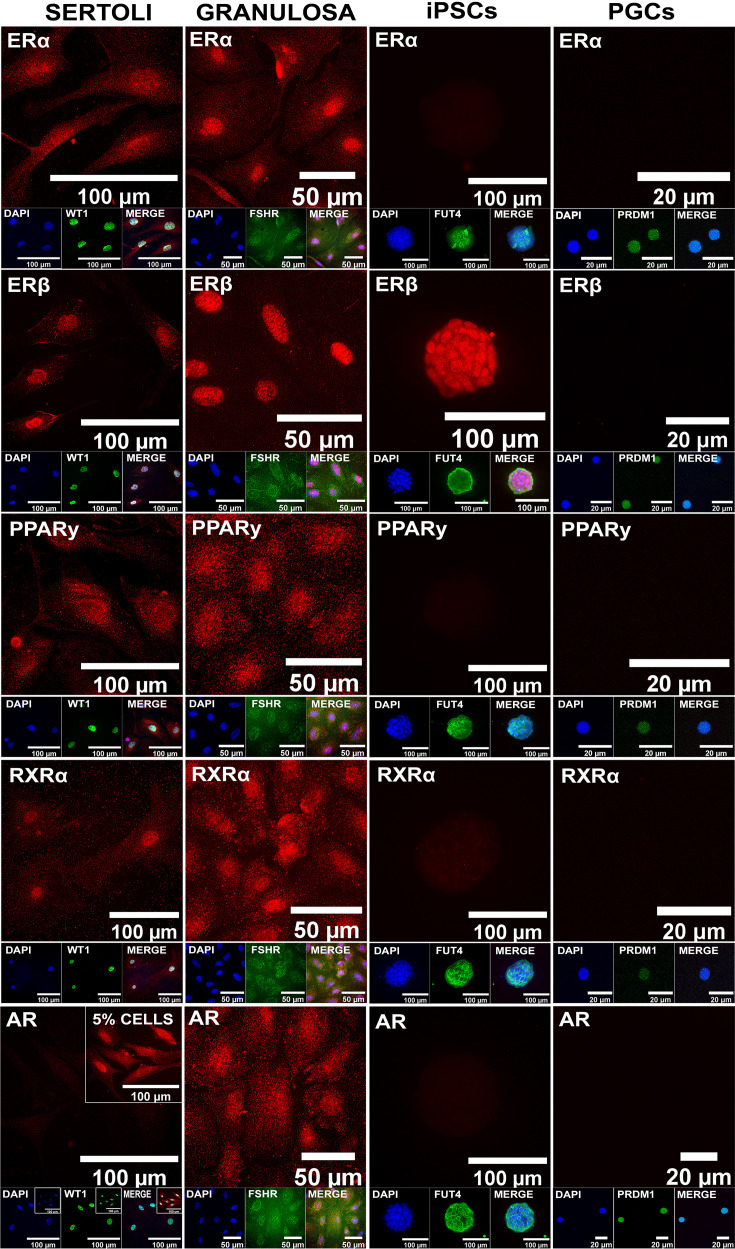
Assessment of expression of additional endocrine receptors potentially involved in cell type-specific responses to BPS exposure. Immunocytochemistry staining of expression of ERα, ERβ, PPARγ, RXRα, and AR is shown, along with staining for known cell-type-specific markers. Somatic cell types express all receptors, pluripotent cells express ERβ but not ERα, PPARγ, RXRα or AR, and germ cells do not express any of the endocrine receptors.

**Figure 3—figure supplement 2.**
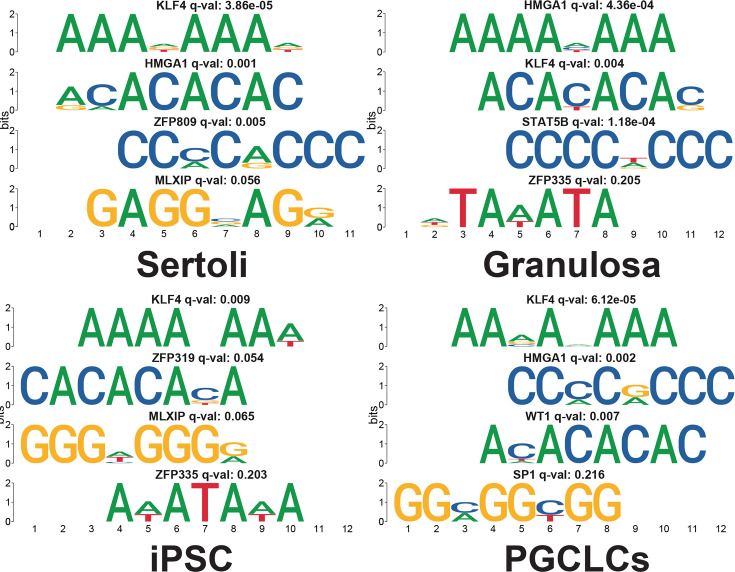
Motifs near enriched DMCs. Identification of the top 4 motif sequences (e-value <0.05) within 500 bp of cell type-specific enriched DMCs that were either associated with enhancer regions in Sertoli, granulosa, and iPS cells or with transcription factor binding sites in PGCLCs. Each motif was compared with the JASPAR database for potential transcription factor binding capability associated with the motif. Transcription factors with potential binding capability are listed above each corresponding motif along with the adjusted p-value (q-value) of the association. Interestingly we see that the two most common motifs across all cell types were associated with either the chromatin remodeling transcription factor HMG1A or the pluripotency factor KLF4.

**Figure 3—figure supplement 3.**
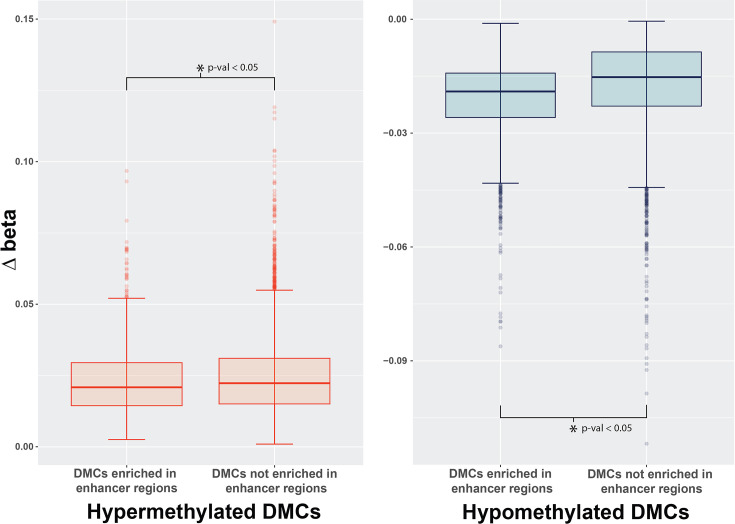
Comparison of delta beta values at significant DMCs. Analysis of the differences in beta values at DMCs from Sertoli, Granulosa, and iPSCs that were enriched at enhancer regions and associated with closer proximity to ERE elements or DMCs that were not enriched at enhancers that had a lower frequency of ERE elements in close proximity. Box plots display a high degree of similarity in the delta beta intensities measured for these specific DMCs in BPS-treated samples vs control samples. Interestingly, the differences between the distribution of beta values were sufficient to be significant based on the two-sample Kolmogorov-Smirnov test. These observed differences indicate that there is higher variability of the delta betas associated with hypomethylated changes occurring at DMCs associated with enhancers but not hypermethylation indicating a trend for a higher proportion of cells to have hypomethylated changes at these specific regions.

**Figure 3—figure supplement 4.**
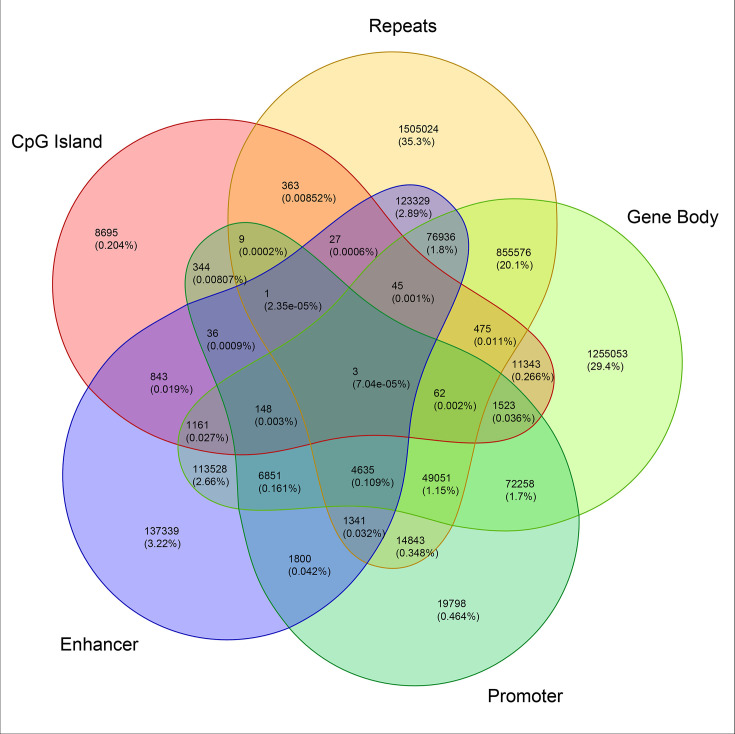
Genome-wide annotation of ERE half-sites. Venn diagram displaying the identification of ERE half-sites localized in known genic and intergenic regions.

These results demonstrate cell-type specific differences in expression of hormone receptors (ERα and ERβ) which are potentially relevant to the disruptive action of the estrogen mimetic, BPS. We noted that expression of at least one potentially relevant hormone receptor (ERβ) in either somatic or pluripotent cells correlated with a higher incidence of DMCs induced by exposure of somatic or pluripotent cell types to 1 µM BPS relative to the incidence of DMCs induced by exposure of PGCLCs, which do not express either estrogen receptor, to the same dose of BPS (Table 2). Nevertheless, we did still observe induction of DMCs when PGCLCs were exposed to BPS, demonstrating that expression of relevant canonical hormone receptors is not an absolute requirement for induction of epimutations in response to an EDC. To exclude the possibility that the observed susceptibility in germ cells could be correlated with the presence of other endocrine receptors that could interact with BPS, we performed additional ICC for the presence of AR, PPARγ, and RXRα and found they were all absent as well in endogenous PGCs (Figure 3—figure supplement 1). This result and our observation noted above that somatic and pluripotent cell types showed a higher incidence of epimutations at apparent enhancer regions while germ cells showed a higher epimutation incidence at apparent promoter regions reveal cell-type specific differences in susceptibility to induction of epimutations by exposure to the EDC, BPS.

### Proximity of BPS-induced epimutations to genomic EREs

The relatively higher incidence of BPS-induced epimutations in cell types expressing one or both estrogen receptor(s) suggests BPS-induced epimutagenesis may be manifest, at least in part, through canonical endocrine signaling pathways. If this is the case, we might expect to see elevated induction of epimutations in genomic regions enriched for relevant HREs which, for interactions with the estrogen mimetic, BPS, would be EREs. Previous studies have defined a full ERE consensus sequence (Bourdeau et al., 2004), but other reports have indicated that estrogen receptors can often bind to ERE half-sites (Mason et al., 2010; Figure 3b). Indeed, when we mined publicly available ERα ChIP-seq peaks from the UCSC genome browser database (Dunham and Kundaje, 2012; Myers et al., 2011; Sloan et al., 2016; Wang et al., 2013; Wang et al., 2012) and performed motif enrichment, we identified two distinct ERE half-sites within regions enriched for DMCs rather than one full-sized ERE consensus sequence, with the half-sites appearing to be more biologically relevant to interaction with estrogen or its mimetics (Figure 3b). We then plotted the frequency of ERE half-sites in genomic regions within 500 bp of BPS-induced epimutations (Figure 3c and d). We found an increase in the frequency of ERE half-sites identified within 500 bp of BPS-induced DMCs genome-wide in all four cell types investigated, but the frequency was notably lower in germ cells (Figure 3c). Thus, this higher frequency of ERE half-sites within 500 bp of BPS-induced DMCs was conserved in three of the four cell types – somatic (Sertoli and granulosa) and pluripotent (iPSCs) when we focused solely on apparent enhancer regions, but not in germ cells (PGCLCs) where the majority of DMCs occurred at apparent promoter regions (Figure 3d). We also mined the UCSC mouse genome sequence for genome-wide prevalence of ERE half-sites and found that while these sites occur in both promoter and enhancer regions, they are nearly fourfold more frequent in enhancers (Figure 3—figure supplement 4 and Table 3). Thus, it is perhaps not surprising that BPS-induced epimutations were more prevalent in enhancer regions in somatic and pluripotent cell types that express one or both ERs, than in germ cells that do not express either ER.

**Table 3.** Summary of ERE annotations.

CpG islands	Repeat regions	Gene bodies	Promoters	Enhancers
25,079	2,631,743	2,448,668	172,707	468,072

Taken together, these results suggest a relationship between (i) expression of either ERβ alone or ERα and ERβ together, (ii) elevated susceptibility to BPS-induced epimutagenesis, and (iii) the occurrence of BPS-induced DMCs in genomic regions – particularly enhancers – containing EREs. These observations are consistent with the notion that one mechanism contributing to EDC-based induction of epimutations involves disruption of canonical endocrine signaling pathways. However, the fact that exposure to BPS also induced epimutations in germ cells, even in the absence of expression of estrogen-related receptors, and generated many DMCs in regions not inclusive of EREs, suggests that disruption of canonical endocrine signaling pathways is not the only mechanism by which exposure to an EDC can induce epimutations.

### Cell-type specific features of BPS-induced epimutations

**Figure 4.**
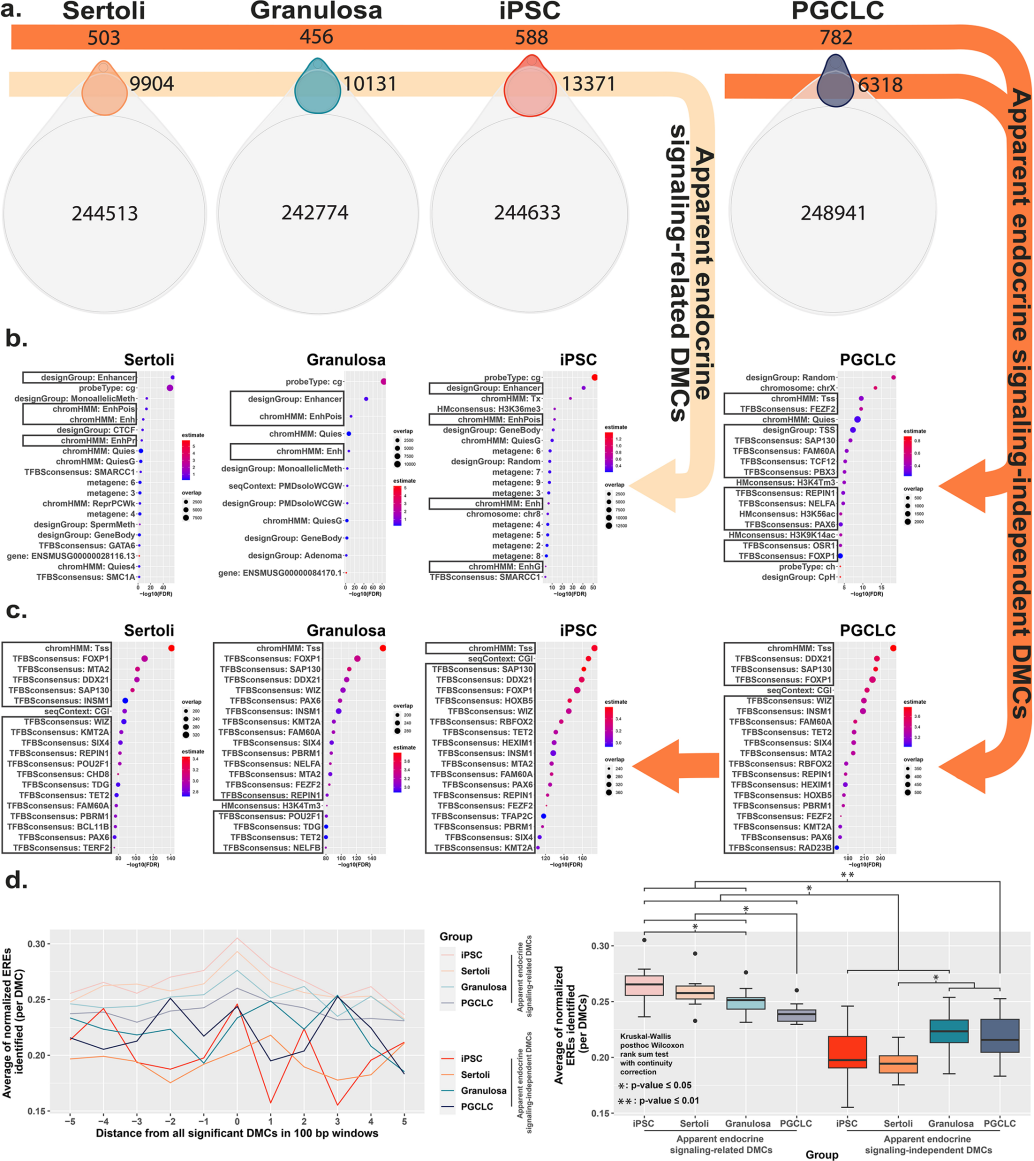
Direct comparison of BPS exposure-specific and cell-type specific features between cell types. (**a**) Assessment Venn diagrams indicating DMCs that are due either to BPS exposure (top, smaller ovals) or inherent cell-type specific differences (bottom, larger ovals). Numbers of apparent endocrine-signaling related DMCs are shown in the light orange arrow, and apparent endocrine-signaling independent DMCs are shown in the dark orange arrows. Enrichment plots indicating feature annotations in genomic regions displaying (**b**) apparent endocrine-signaling related DMCs occurring predominantly in enhancer regions in somatic Sertoli and granulosa cell types or pluripotent cells expressing one or more estrogen receptors, or (**c**) a smaller set of apparent endocrine-signaling independent DMCs occurring predominantly in promoter regions in all four cell types regardless of +/-expression of relevant endocrine receptors. (**d**) Normalized density plots and box plots displaying the frequency of ERE half-sites identified within 500 bp of apparent endocrine-signaling related DMCs occurring predominantly in enhancer regions and apparent endocrine-signaling independent DMCs occurring predominantly in promoters.

To further interrogate cell-type specific differences in the genesis of epimutations following exposure to BPS, we compared DNA methylation patterns detected in the control (vehicle only) samples to identify naturally occurring, cell-type specific DMCs inherently associated with each different cell fate. Interestingly, of the 297,415 distinct CpGs interrogated by the Illumina Infinium Mouse Methylation BeadChip Array used for this analysis, >240,000 showed some degree of inherent differential methylation among the four cell types tested, suggesting they are tied to cell-fate specific differential DNA methylation (Figure 4a). We next determined the extent to which DMCs induced specifically by exposure of each cell type to BPS occurred at CpG dinucleotides that were among these naturally occurring cell-type specific DMCs. We found that a large majority of BPS exposure-specific epimutations detected in each cell type occurred at CpGs that also show inherent, cell-type specific variation in DNA methylation (>95% of BPS-induced epimutations in somatic and pluripotent cell types and ~89% in germ cells; Figure 4a). As with the overall pattern of BPS-induced epimutations shown in Figure 2, BPS-induced epimutations at CpGs showing inherent cell-type specific variation were enriched in apparent enhancer regions that occurred near EREs in somatic and pluripotent cell types, whereas those in germ cells were found predominantly in promoter regions containing significantly fewer EREs (Figure 4b and d). However, the low percentage of BPS-induced epimutations that occurred at CpGs that did not show inherent cell-type specific variation in DNA methylation patterns were found to be enriched in promoter regions lacking nearby EREs in all four cell types (Figure 4c and d). Thus, this latter group of BPS-induced epimutations appears to represent a small core group that arises following exposure to BPS via a mechanism that does not rely upon disruption of canonical endocrine signaling, and so is common to all cell types, regardless of expression of relevant endocrine receptors or nearby residence of relevant HREs within the genome.

### Impact of BPS exposure on gene expression

We next sought to determine if the cell-type specific differential susceptibility to BPS-induced epimutagenesis translated to a similar extent of dysregulation of gene expression in each cell type. We found that there was a similar relationship between the presence of DMCs in the promoter region of a gene and differential expression of that gene in all four cell types examined (Figure 1—figure supplement 2b). Somewhat surprisingly, RNA-seq analysis of gene expression patterns in each exposed cell type relative to its corresponding control (same cell type exposed to carrier only) revealed the greatest number of dysregulated genes in PGCLCs, despite PGCLCs being the cell type that showed the lowest number of epimutations following exposure to BPS (Tables 2 and 4). iPSCs showed the second highest level of dysregulated genes, while somatic Sertoli and granulosa cells showed relatively low levels of dysregulated gene expression (Table 4). Indeed, numbers of dysregulated genes were three orders of magnitude lower in differentiated somatic cells than in either pluripotent cells or germ cells (Table 4).

**Table 4.** Exposure-specific differentially expressed genes^*^.

DEGs	Sertoli	Granulosa	iPSCs	PGCLCs
Down-regulated	3	0	343	844
Up-regulated	32	2	694	1046
Total	35	2	1037	1890

* Genes showing significant differential expression following exposure of each cell type to 1 µM BPS relative to matched control cell types exposed to carrier only.

**Figure 5.**
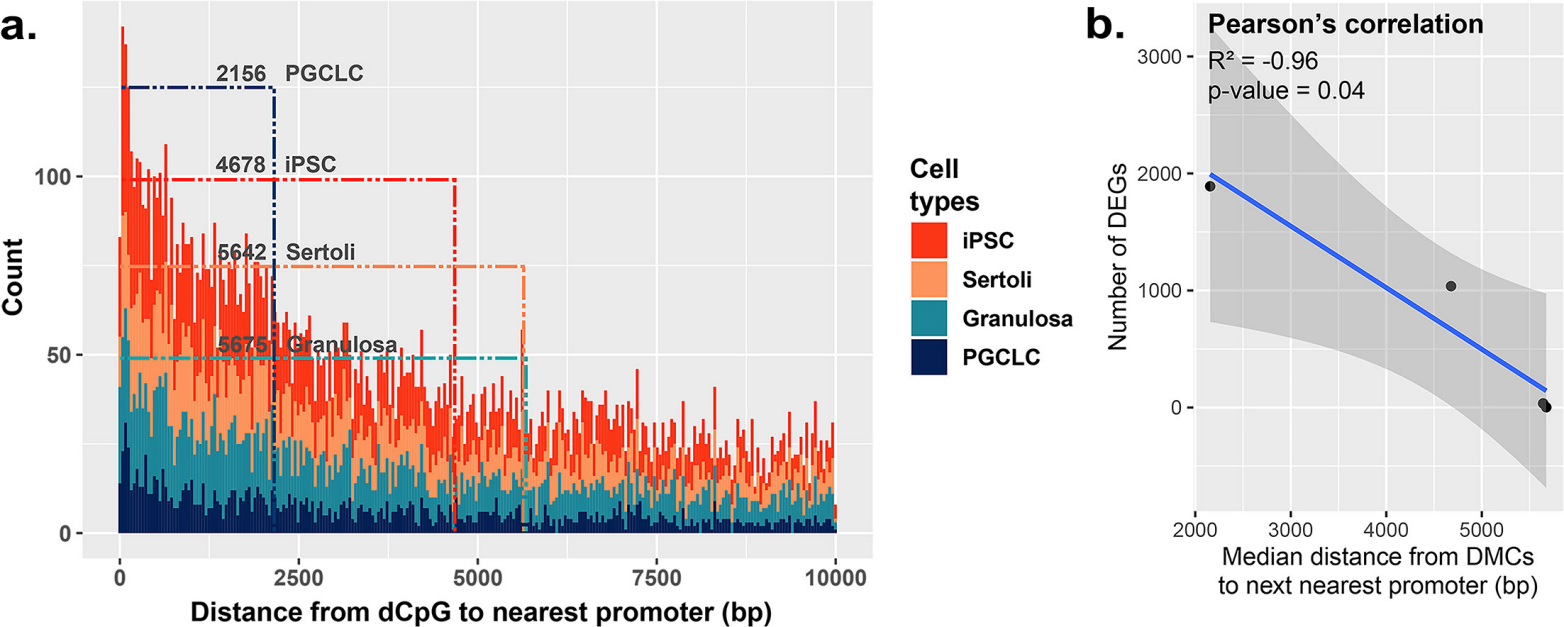
Correlation between the proximity of DMCs to promoters and dysregulation of gene expression. (**a**) Proximity plot displaying distances from exposure-specific DMCs to nearest promoter regions. Dotted lines indicate median points of the data for each cell type. (**b**) Correlation plot displaying a negative relationship between the distance from DMCs to nearest promoters and resulting dysregulation of gene expression within each cell type.

Because BPS-exposed PGCLCs showed the highest level of dysregulated genes, as well as the highest enrichment of DMCs occurring primarily at promoters (Figure 2b), we next assessed the general proximity between DMCs and promoter regions in each cell type to determine if DMCs in promoter regions may be more likely to predispose dysregulated gene expression than DMCs elsewhere in the genome (Figure 5a). Indeed, we found that in all four cell types, the smaller the median distance between BPS-induced DMCs and neighboring promoter regions, the larger the number of BPS-induced DEGs (Figure 5b). Thus, it appears that while PGCLCs showed the fewest overall BPS-induced DMCs among the four cell types exposed to BPS, this exposure induced a higher proportion of epimutations in regions within or adjacent to promoters in this cell type. This, and the relatively high extent of decondensed chromatin genome-wide in fetal germ cells, appear to have contributed to the higher incidence of dysregulated gene expression in BPS-exposed PGCLCs than that observed in the other three BPS-exposed cell types, even though the overall numbers of BPS-induced epimutations were greater in the other cell types.

**Figure 5—figure supplement 1.**
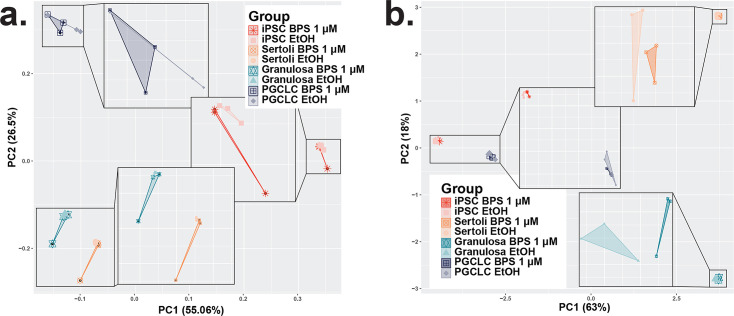
Consistency among replicates of pluripotent, somatic, and germ cell types and variation between DNA methylation Infinium Beadchip array experimental and control groups. Plots displaying principle component analyses of data from the cell-type-specific susceptibility to BPS via changes in DNA methylation from DNA methylation Infinium Beadchip array data and (**d**) gene expression from bulk RNA-seq data. The dimensional reduction of the variation in plots displays that replicate samples cluster into regions based on distinct cell identity profiles with only minimal overlap between treatment and control samples.

### Potential non-canonical signaling pathways disrupted by BPS exposure

**Figure 6.**
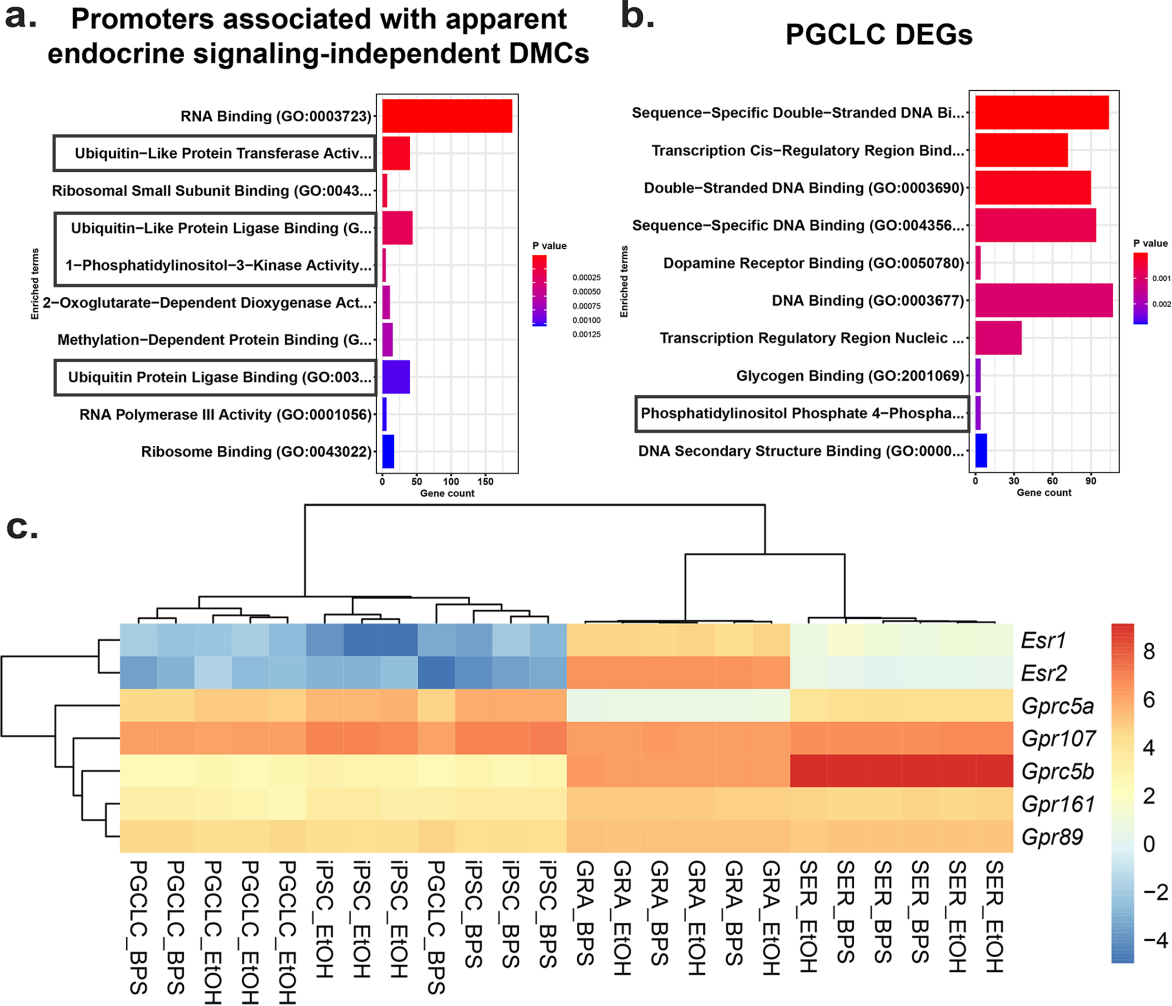
Potential involvement of non-canonical estrogen signaling pathways in BPS-induction of epimutations. Relative expression of genes (**a**) enriched for apparent endocrine-signaling independent promoter-region DMCs found in all cell types or (**b**) dysregulated in PGCLCs which lack expression of estrogen receptors. (**c**) Heatmap of relative expression of estrogen receptor genes (*Esr1* and *Esr2*) and G-coupled protein receptors (*Gprc5a*, *Gpr107*, *Gprc5b*, *Gpr161*, and *Gpr89*) in pluripotent, somatic, and germ cell types. *Gprc5a*, *Gpr107*, *Gprc5b*, *Gpr161*, and *Gpr89* all have been shown to bind to BPA or 17β-estradiol in rat models and represent potential G-coupled protein receptors which could lead to the induction of endocrine-signaling independent DMCs.

To identify potential mechanisms by which BPS exposure may induce epimutations via disruption of non-canonical endocrine signaling pathways, we mined our RNA-seq data to identify genes dysregulated independently of expression of relevant hormone receptors or presence of nearby EREs. We identified a set of genes in all four cell types tested that displayed promoters enriched for apparent endocrine-signaling independent DMCs (Figure 6—figure supplement 1). Gene ontology (GO) analysis indicated that several of the 1957 genes we identified were associated with ubiquitin-like protease pathways, including 124 of 800 genes that regulate protein degradation and 5 of 9 genes involving 1-phosphatidylinositol-3 kinase activities, linked to the PI3K/AKT signaling pathway (Figure 6a).

**Figure 6—figure supplement 1.**
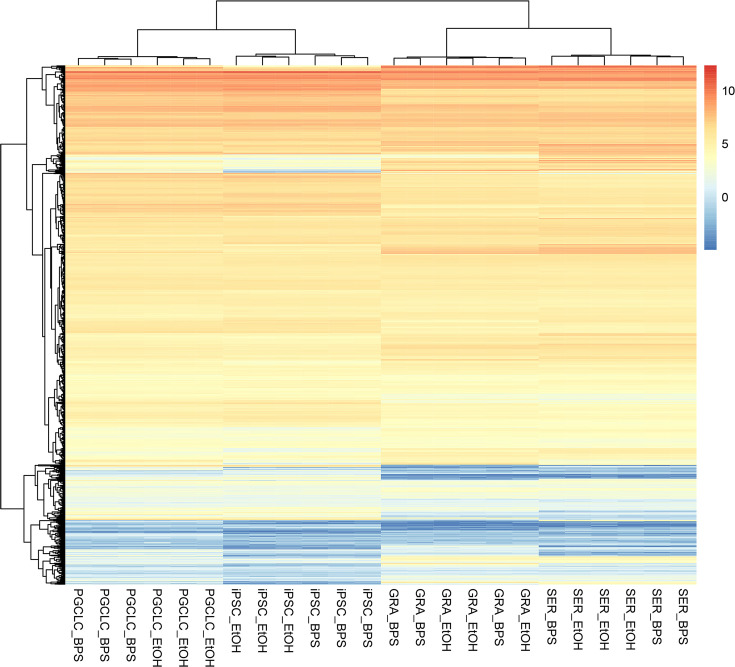
Differential expression of potential endocrine-signaling independent DMCs. Heatmap displaying the relative expression of genes with promoters enriched for apparent endocrine-signaling independent DMCs. The majority of these genes displayed a similar pattern of active expression in all cell types examined.

Previous reports have established a link between ubiquitin-like protease pathways and classical ER signaling (Beamish and Frick, 2022; Kabir et al., 2015), suggesting that genes related to ubiquitin-like protease pathways that have promoters lacking EREs may be regulated by factors that are themselves encoded by genes with promoters containing EREs, and so could be indirectly activated or repressed by disruption of classical ER signaling. However, involvement of PI3K/AKT pathway signaling has previously been linked to estrogen signaling through non-canonical G-protein coupled receptors such as GPER1, which was sufficient to induce estrogen signaling in ER KO mouse cell lines which lack the capacity for classical ER signaling (Filardo et al., 2002; Molina et al., 2017). To determine if our data support the suggestion that the non-canonical BPS-induced changes were linked to involvement of PI3K/AKT pathway genes, we performed GO analysis on the 1890 DEGs identified in PGCLCs in which epimutations appeared to be induced independent of classical ER signaling. Interestingly, we detected no apparent involvement of pathways involving ubiquitin-like proteases, but we did detect differential expression of four of seven genes associated with a pathway involving phosphatidylinositol-4 phosphatase signaling which intersects with the PI3K/AKT signaling pathway (Figure 6b). Finally, while we did not detect expression of *Gper1* transcripts, we did observe differential expression of genes encoding other less well studied G-protein-coupled receptors, including *Gprc5a*, *Gprc5b*, *Gpr89, Gpr107*, and *Gpr161* in all four cell types exposed to BPS. These receptors have all been previously shown to bind either BPA or 17β-estradiol in rat models and could be potential targets of non-canonical BPS-induced estrogen signaling (Figure 6c) as described in the following links: (Rat Genome Database, 2024a; Rat Genome Database, 2024b; Rat Genome Database, 2024c; Rat Genome Database, 2024d; Rat Genome Database, 2024e).

### Propagation of BPS-induced epimutations during transitions in cell fate

**Figure 7.**
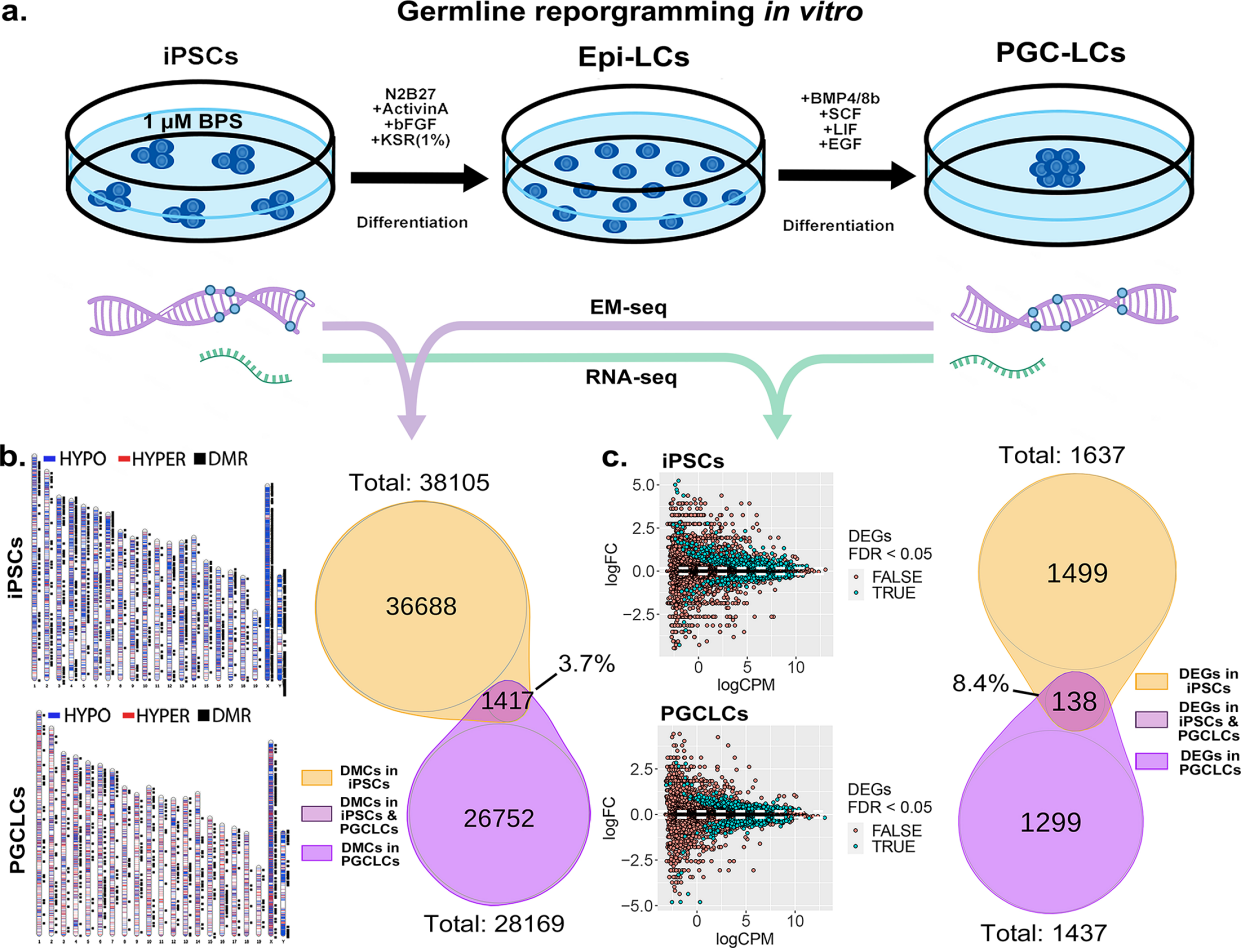
Persistence of BPS-induced epimutations through recapitulation of early germline reprogramming in vitro. (**a**) Schematic illustrating derivation of PGCLCs from iPSCs in vitro. iPSCs are first induced to form EpiLCs which are then induced to form PGCLCs. iPSCs were exposed to either ethanol +1 μM BPS or ethanol (carrier) only, then induced to undergo transitions to form EpiLCs and then PGCLCs. (**b**) DNA samples from BPS-exposed or control iPSCs as well as subsequently derived PGCLCs were assessed for exposure-specific DNA methylation epimutations by EM-seq. BPS-treated iPSCs showed 38,105 DMCs and subsequently derived PGCLCs showed 28,169 DMCs. Of those, only 1417 (3.7%) of the DMCs were conserved from the BPS-exposed iPSCs to the subsequently derived PGCLCs. (**c**) RNA samples from BPS-exposed or control iPSCs and subsequently derived PGCLCs were assessed for global gene expression patterns by RNA-seq. BPS-treated iPSCs showed 1637 exposure-specific DEGs and subsequently derived PGCLCs showed 1437 exposure-specific DEGs. Of those, only 138 (8.4%) were conserved from the BPS-exposed iPSCs to the subsequently derived PGCLCs. Figure 7—source data 1.Quality control metrics for EM-seq data.

Transitions between pluripotent and germ cell fates, or vice versa, are accompanied by large-scale epigenetic reprogramming in vivo (Cantone and Fisher, 2013; Lee et al., 2014; Santos et al., 2002; Sanz et al., 2010), and these are recapitulated during similar transitions induced in vitro (Ishikura et al., 2016). To determine the extent to which BPS-induced epimutations persist during a pluripotent to germline transition in vitro, we first exposed iPSCs to 1 μM BPS and then differentiated the exposed iPSCs first into epiblast-like cells (EpiLCs) and then into PGCLCs, recapitulating the early germline epigenetic reprogramming event that normally occurs in vivo (Kurimoto and Saitou, 2018; Ohta et al., 2017; Figure 7a). We then used genome-wide analyses by EM-seq and RNA-seq to compare numbers of exposure-specific DMCs and DEGs, respectively, in PGCLCs derived from BPS-exposed iPSCs with those in the directly exposed iPSCs and found lower, but still substantial numbers of both (28,168 vs 38,105 DMCs, and 1437 vs 1637 DEGs in the derived PGCLCs compared to the directly exposed iPSCs, respectively; Figure 7b and c).

**Figure 7—figure supplement 1.**
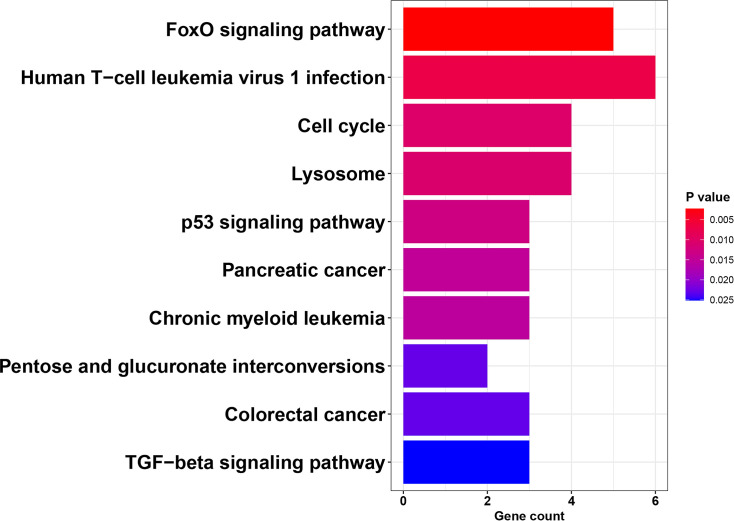
KEGG pathway analysis of DEGs detected in both iPSCs exposed to BPS and PGCLCs derived from the exposed iPSCs. Analysis of KEGG pathways associated with 138 BPS-induced DEGs that persisted during the transition in cell fate from BPS-exposed iPSCs to PGCLCs revealed genes primarily involved with cell cycle and apoptosis pathways.

**Figure 7—figure supplement 2.**
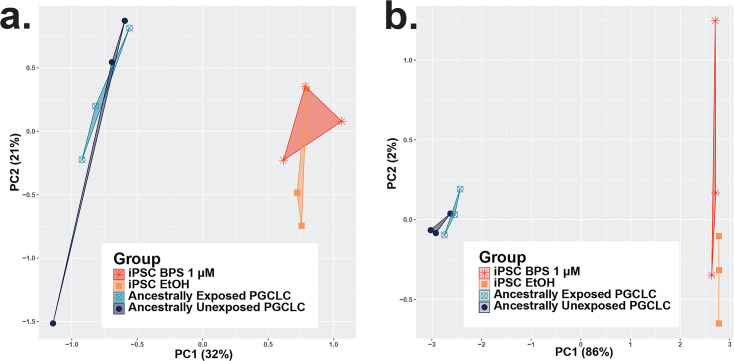
Consistency among iPSC and ancestrally exposed PGC-LC replicates and variation between RNA-seq and EM-seq experimental and control groups. Plots displaying principle component analyses of data from the persistence of epimutations through transitions in cell states based on (**a**) DNA methylation from EM-seq data and (**b**) gene expression from bulk RNA-seq data. Again, replicate samples clustered into regions based on distinct cell identity profiles. However, there is a lack of strong separation between treatment conditions in the second principle component. While the differences in all experiments were sufficient to produce DMCs/DMRs/DEGs, the separation between treatment conditions displayed by the PCA could likely be increased by a larger sample size and indicate a limitation of only having triplicate replicates for this study.

**Figure 7—figure supplement 3.**
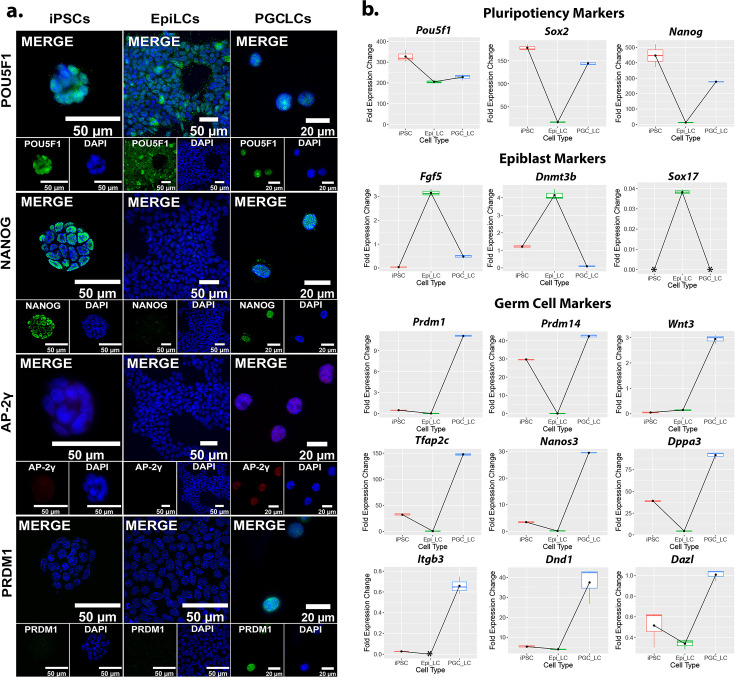
Relative expression of markers for PGCLC induction from iPSCs. (**a**) ICC of pluripotency and germ cell marker expression throughout the transition from iPSCs to PGCLCs. (**b**) qRT-PCR of pluripotency, epiblast, and germ cell markers indicating gene expression profiles during induction of PGCLCs from iPSCs. Each gene fold expression is relative to the housekeeping gene *Gusb* using the ∆Cq method. The symbol * indicates that the expression of transcripts in the sample was either non-existent or so low as to be undetectable by qRT-PCR.

**Figure 7—figure supplement 4.**
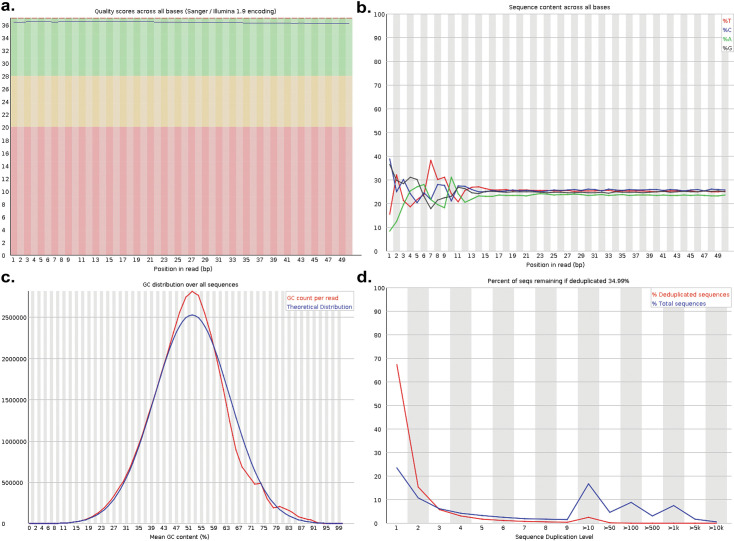
Quality control for RNA-seq data. RNA-seq quality control data from one of the three PGCLC replicates exposed to BPS as an example. (**a**) Base calls showed high-quality scores (phred scores >30) for all bases in reads. (**b**) Reads showed equal distributions of all four bases following the initial adaptor sequence and sufficient base complexity. (**c**) Distribution of GC sequences across reads aligned very closely with the theoretical distribution. (**d**) Duplication plot indicates deduplicated libraries contained ~67% unique sequences which indicates sufficient library complexity for subsequent downstream data processing.

When we compared the specific DMCs and DEGs that were detected in the BPS-exposed iPSCs with those that were detected in the subsequently derived PGCLCs, we found that >90% of each were not conserved during the pluripotent to germline transition in cell fate. Specifically, only 3.7% of the DMCs and 8.4% of the DEGs detected in the BPS-exposed iPSCs were also detected in the PGCLCs derived from the exposed iPSCs. Among the small portion of specific 138 DEGs (Supplementary file 1) that were conserved from exposed iPSCs to derived PGCLCs, several (12.32%) (*Cdkn1a*, *Ccnd2*, *Plk2*, *Tgfbr1*, *Gadd45g*, *Lck*, *Ltbr*, *Mad2l1*, *Ap3m2*, *Ctsz*, *Tcirg1*, *Gusb*, *Id2*, *Lefty2*, *Gstm*7*, Acsl1, Slc39a14*) were involved in cell cycle and apoptosis pathways which could potentially be linked to cancer development (Figure 7—figure supplement 1). Thus, of the 38,105 DMCs and 1637 DEGs induced by exposure of iPSCs to 1 µM BPS, 36,688 and 1499, respectively, did not persist during differentiation of iPSCs to form PGCLCs, and so were apparently corrected by germline reprogramming. Simultaneously however, 26,752 novel DMCs and 1299 novel DEGs appeared in the derived PGCLCs that were not present in the BPS-exposed iPSCs, so were apparently generated de novo during the germline reprogramming process. These results are consistent with the notion that germline epigenetic reprogramming corrected many of the epimutations that were present in the BPS-exposed pluripotent cells from which they were derived, but that exposure of cells to EDCs may disrupt the underlying chromatin landscape in a way that then interferes with subsequent reprogramming such that in addition to correcting many previously induced epimutations, the germline reprogramming process, acting on a disrupted chromatin landscape, also generates many novel epimutations de novo during the pluripotent to germline transition.

## Discussion

While nearly 20 years of research on the effects of exposure of live animals to various EDCs and other environmental disruptive influences has clearly established the potential to perturb the epigenome in ways that can dysregulate normal gene expression patterns and predispose the development of disease states, the molecular mechanisms underlying these phenomena have remained largely undefined. Thus, the manner in which environmental exposures introduce biochemical changes in epigenetic programming, how such disruptions can be propagated within a tissue or throughout the soma of an exposed individual or enter that individual’s germ line, or how these disruptions predispose disease states in that individual, or how a resulting prevalence of epimutations can be transmitted to multiple subsequent generations despite generational epigenetic reprogramming, are all mechanistic questions that have remained unanswered despite more than 5000 publications on this topic since the early 2000s. The vast majority of those publications have described experiments in animal models – typically rodents. While informative, such live animal studies require months to years to complete, are cost-, labor-, and animal-intensive, and have not yielded substantial insight into the molecular mechanisms underlying the deleterious effects of environmentally induced epimutations.

In vitro model systems have proven to be very useful tools for deciphering molecular mechanisms related to normal development and homeostasis, or disruptions of those processes predisposing onset of disease. Examples include cell culture systems used to study developmental processes such as X-chromosome inactivation in mammals (Almeida et al., 2017; Patrat et al., 2009) or early embryogenesis (Bao et al., 2022; Lau et al., 2022), or to elucidate the cellular and molecular etiology of many different diseases based on the now popular ‘disease-in-a-dish’ approach (Davaapil et al., 2020; Song et al., 2023). While in vitro cell culture systems are, by nature, devoid of a majority of the physiological complexities present within the intact organism in vivo, this provides an actual advantage in that it facilitates deconvolution of those complexities while unequivocally focusing on only certain, specific aspects or cell types. However, this ultimately warrants validation of results discerned from studies of in vitro models to ensure they also reflect functions ongoing in the more complex and heterogeneous environment of the intact animal in vivo.

We assembled an in vitro system in which we combined the three cell types normally involved in (i) the initial exposure to disruptive environmental effects (somatic cells), (ii) the first phase of generational epigenetic reprogramming in the preimplantation embryo (pluripotent cells), and (iii) the second phase of generational reprogramming in the developing germ line (germ cells). This system allowed us to gain insights into multiple mechanistic aspects of environmentally induced epimutagenesis including the initial induction of epimutations by exposure of cells to the EDC, BPS, cell-type specific differences in susceptibility to BPS-induced epimutagenesis, factors that appear to contribute to that differential susceptibility, and the mechanism by which an epimutated state may persist across multiple generations despite generational reprogramming.

Here we have shown that exposure of homogeneous populations of cells maintained in culture to an EDC such as BPS can induce epimutagenesis and dysregulation of gene expression, and that this occurs in a partially additive dose-dependent manner, although the quantitative effect appears to plateau at higher doses. This afforded us the opportunity to test the relative effects of direct exposure of different specific cell types to a similar dose of BPS with no confounding effects imposed by interactions with other cell types or systemic effects of the sort that normally occur in the in vivo context. We found that somatic, pluripotent, and germ cell types do indeed show differences in direct susceptibility to induction of epimutations following exposure to BPS, both in terms of the quantity of epimutations induced and qualities of the resulting epimutations such as a predominance of hyper- versus hypomethylation, and the genomic locations where such epimutations tend to occur. Our results suggest that these differences reflect distinctions in the normal status of each cell type at the time of exposure. Thus, somatic, pluripotent, and germ cell types differ in the normal status of genome-wide patterns of accessible versus inaccessible chromatin, global DNA methylation, and expression of relevant endocrine receptors. Interestingly, our data suggest that all of these parameters can influence cell-type specific susceptibility to induction of epimutations by exposure to BPS, and that different combinations of these variables ultimately determine the quantity and quality of epimutations induced by exposure to BPS. We note that while our exposure dose of 1 µM of BPS was below that deemed safe by the EPA for exposure of humans to the similar EDC, BPA, that same dose may exert greater effects when used to expose cells in culture in the absence of any sort of mitigating metabolic effects that may accrue in intact animals or humans. Indeed, the potential to quantify the epimutagenic effects of different doses of an EDC on different cell types as shown in our study could be used in future studies to assess the relative effects of a specific dose of an EDC on a specific cell type when that cell type is exposed either in a homogeneous culture or within an intact animal.

Endogenous fetal germline cells (e.g. PGCs and prospermatogonia in males) normally display lower levels of global DNA methylation and higher levels of chromatin accessibility than pluripotent or somatic cell types (Hajkova, 2011), yet PGCLCs developed fewer epimutations following exposure to BPS than the other cell types. This appears to be explained by the fact that germline cells do not express either estrogen receptor, whereas pluripotent and somatic cell types express one or both of the two estrogen receptors, apparently rendering them more susceptible to induction of epimutations following exposure to the estrogen mimetic, BPS. This is consistent with the notion that EDCs impose disruptive effects by interfering with canonical endocrine signaling pathways, and that notion is further supported by our observation that, in the cell types that express one or both estrogen receptors, we observed a prevalence of BPS-induced epimutations in genomic regions containing nearby EREs, whereas in PGCLCs, which do not express either estrogen receptor, we did not observe a strong correlation between the locations of BPS-induced epimutations and genomic EREs.

Nevertheless, we did observe induction of epimutations in PGCLCs exposed to BPS, suggesting that while cell types expressing relevant endocrine receptors may display elevated susceptibility to EDC-induced epimutagenesis presumably based on disruption of canonical endocrine signaling, this is not the only mechanism by which EDCs can induce epimutations. Indeed, our results support the suggestion that there may be at least two types of epimutations induced by exposure to EDCs – one that is independent of canonical endocrine signaling and so occurs in all cell types regardless of expression of relevant endocrine receptors or the genomic location of relevant HREs, and another that does involve disruption of canonical endocrine signaling and so is elevated in cell types expressing relevant endocrine receptors and predisposes the induction of epimutations in genomic regions near relevant HREs. Beyond this, a comparison of the quantities of BPS-induced epimutations in pluripotent and somatic cell types, which both express one or both estrogen receptors, but which differ with respect to elevated genome-wide chromatin accessibility in pluripotent cells compared to much more limited chromatin accessibility in somatic cell types (Cantone and Fisher, 2013; Santos et al., 2002), suggests that chromatin accessibility also contributes to susceptibility to EDC-induced epimutagenesis.

One of our most intriguing findings was that, in addition to estrogen-receptor expressing somatic and pluripotent cell types developing more epimutations than non-estrogen-receptor expressing germ cells in response to exposure to BPS, the occurrence of epimutations in the former (somatic and pluripotent) cell types was predominantly in enhancer regions whereas that in the latter (germ) cell type was predominantly in gene promoters. As noted above, there was a significant increase in the occurrence of BPS-induced epimutations near EREs in somatic and pluripotent cell types compared to germ cells. A genome-wide analysis confirmed that ERE half-sites occur more frequently in enhancer regions than in promoter regions (Table 3 and Figure 3—figure supplement 4) supporting our suggestion that the higher prevalence of BPS-induced epimutations we observed in enhancer regions in somatic and pluripotent cell types was due to disruption of canonical hormone signaling.

Our finding that PGCLCs, which do not express endocrine receptors, still accrued epimutations following exposure to BPS indicates that in addition to inducing epimutations by disrupting canonical endocrine signaling pathways, exposure to EDCs can induce epimutations via mechanisms that do not involve disruption of such pathways. This notion is further supported by the occurrence of BPS-induced epimutations in genomic regions that do not include nearby EREs, even though a prevalence of the epimutations induced in PGCLCs were located in gene promoters. Pathway analysis of the genes in which promoter-region epimutations were induced by exposure of PGCLCs to BPS suggests an apparent endocrine-signaling independent group of genes that can become disrupted by exposure to an EDC via non-canonical signaling which may occur through the PI3K/AKT pathway via G-protein-coupled receptors. While we observed expression of a number of G-protein-coupled receptors known to bind to either 17β-estradiol or BPA, further studies will be required to determine if any of these contribute to susceptibility to BPS epimutagenesis.

As expected, disruption of normal epigenetic programming induced by exposure of cells to BPS also led to dysregulation of gene expression. Thus, RNA-seq analysis detected differentially expressed genes in all cell types exposed to BPS. Surprisingly, the greatest number of DEGs was detected in PGCLCs which was the cell type that developed the lowest overall number of epimutations in response to exposure to BPS. However, PGCLCs showed a higher prevalence of BPS-induced epimutations in gene promoter regions than was observed in any of the other cell types, so it appears that promoter-region epimutations impose the most direct impact on gene expression. As noted above, fetal germ cells normally display the lowest level of global DNA methylation of any cell type at any developmental stage. This is characteristic of the epigenetic ground state that accrues uniquely in developing germ cells (Hajkova, 2011), and is believed to reflect a generally more accessible chromatin state genome-wide in germ cells than in other cell types. Thus, it may be that the epigenetic ground state in developing germ cells renders this cell type uniquely susceptible to epimutagenesis in gene promoter regions, thereby predisposing a high level of dysregulation of gene expression in response to exposure to an EDC.

Finally, our in vitro system allowed us to follow the fate of BPS-induced epimutations through a major transition in cell fate from pluripotent to germline cells during which a portion of the normal generational germline epigenetic reprogramming process is known to occur (Kurimoto and Saitou, 2018). Thus, when we induced iPSCs previously exposed to 1 μM BPS to differentiate into PGCLCs, we found that a substantial prevalence of epimutations persisted during this transition. However, very few of the specific DMCs or the associated DEGs detected in the BPS-exposed iPSCs persisted in the subsequently derived PGCLCs. Specifically, only 3.7% of DMCs and 8.4% of DEGs were conserved between the BPS-exposed iPSCs and the subsequently derived PGCLCs. This suggests that exposure of cells to EDCs has the potential to disrupt epigenetic programming in a way that then interferes with subsequent generational reprogramming that normally accompanies either germline to pluripotent or pluripotent to germline transitions in cell fate. Recent in vivo studies have suggested that exposure of gestating female mice to the EDC tributyltin results in disruption not simply of the pattern of epigenetic modifications but also of the underlying chromatin landscape (Chamorro-García et al., 2021; Chang et al., 2022). In this context, our results are consistent with the suggestion that exposure of cells to EDCs can disrupt the underlying chromatin landscape (e.g. the pattern of A and B chromatin compartments) such that when the ‘normal’ reprogramming machinery then acts on this disrupted landscape it corrects many of the originally induced epimutations, but simultaneously induces many novel epimutations de novo. This would predispose ongoing abnormalities in the underlying chromatin landscape that would, in turn, lead to the recurring correction of many existing epimutations in concert with the genesis of many novel epimutations during each subsequent generation.

We note that, with the exception of our analysis of granulosa cells, all of our studies were carried out in ‘male’ XY-bearing cells. It remains possible that XX-bearing cells might differ from XY-bearing cells in the way(s) in which they respond to exposure to an EDC. However, the similarity we observed between responses of XX granulosa cells and XY Sertoli cells suggest this may not be the case – at least in cell types in which dosage compensation has been established by X-chromosome inactivation.

Taken together, our results demonstrate the utility of an in vitro cell culture approach for pursuing molecular mechanisms underlying environmentally induced disruption of normal epigenetic programming manifest as the formation of epimutations and dysregulated gene expression. This approach clearly affords unique potential to reveal mechanisms responsible for the initial induction of epimutations in response to direct exposure of cells to disruptive effects such as EDCs, as well as offering potential means to elucidate mechanisms by which environmentally induced epimutations are then propagated within the exposed individual and then to that individual’s descendants. Knowledge of these mechanisms will afford the best opportunity to understand how these defects may, in the future, be better diagnosed, treated, and/or prevented. With the ever-expanding catalog of potentially hazardous man-made compounds permeating our environment, it is increasingly important that we recognize the potential dangers such compounds may impose and maximize our ability to protect ourselves from those dangers.

## Materials and methods

### Animal procedures

Mice were used as the primary source of cells for the establishment of cultures for the analysis of differential cell-type specific susceptibility to exposure to the EDC BPS at the cellular level. All mice were euthanized prior to dissection and isolation of the tissue or cell type to be used for experiments. All animals were bred on-site and maintained in one of the UTSA on-campus vivaria under controlled temperature and humidity conditions in a fixed 12 hr light, 12 hr dark cycle with free access to 5V5R extruded food and non-autoclaved conventional RO water. 5V5R has been verified to contain a targeted level of 50 PPM total phytoestrogenic isoflavones (genistein, daidzein, and glycitein) and has been certified for use with estrogen-sensitive protocols limiting the effect of these plant-derived compounds. Euthanasia was performed by trained personnel using continuous CO_2_ exposure at a rate of 3 L/min until one minute after breathing had stopped. Euthanasia was confirmed by cervical dislocation. Tissue from euthanized mice was used to obtain somatic (Sertoli, granulosa, and mouse embryonic fibroblast [MEF]) cells, and primordial germ cells [PGCs]. Both Sertoli and granulosa cells were isolated from respective male and female mice euthanized at postnatal day 20 (P20). MEFs and PGCs were isolated from fetuses at embryonic day 13.5 postcoitum (E13.5).

### In vitro generation and/or culture of pluripotent, somatic, and germ cells

#### Pluripotent cell culture

Male mouse iPSCs were derived from a transgenic mouse line carrying the *tetO-4F2A* cassette obtained from The Jackson Laboratories (011011) (The Jackson Laboratory, ME USA). iPSCs can be induced from essentially any cell type carrying this reprogrammable cassette by exposure to Doxycycline for one week as previously described (Carey et al., 2010; Hochedlinger et al., 2005). For this project, iPSCs were reprogrammed from MEFs isolated from a single male (XY) E13.5 mouse fetus. Reprogrammed colonies were picked and expanded via sub-passaging. Validation of reprogrammed pluripotency was determined by positive immunocytochemical (ICC) staining for the pluripotent markers POU5F1, SOX2, NANOG, and FUT4 (Figure 1—figure supplement 5 and Figure 2—figure supplement 2). Karyotyping was done on each candidate iPSC line produced to confirm normal chromosome number and XY sex chromosome constitution of each line prior to aliquots being prepared for long-term storage in liquid nitrogen (Supplementary file 2). We selected a male (XY) iPSC line to be used for this project. Upon thawing, iPSCs were initially maintained on CF-1 feeder cells for a minimum of three passages. Cells were cultured in DMEM supplemented with 15% fetal bovine serum (FBS) and 1000 U/mL leukocyte inhibitory factor (LIF). Once pluripotent cells were stabilized in culture, they were transitioned to feeder-free conditions and were cultured in N2B27 media supplemented with 2i and LIF for two-three passages prior to use in chemical exposure experiments. A complete list of the media components along with catalog numbers can be found in Supplementary file 3.

#### Sertoli cell culture

Primary cultures of Sertoli cells were established as previously described (Karl and Griswold, 1990). Briefly, whole testes were dissected from ~5 juvenile littermate mice at P20. Following removal of the tunica albuginea from each individual testis, the bundles of seminiferous tubules were physically chopped using a sterile razor blade to break down the coiled structure of tubules increasing the surface area and rendering them easier to digest. These shortened fragments of seminiferous tubules were then digested in a mixture of 2.5% trypsin and 6.64 mg/ml DNaseI in DPBS for 25 min at 37 °C. After these enzymes were inactivated, the tubule sections were washed multiple times, then treated with a final enzymatic mixture of collagenase IV (0.70 mg/mL) and DNaseI (6.64 mg/mL) for 10 min to permeabilize the thick collagen layer on the exterior of the seminiferous tubules to allow the Sertoli cells to migrate out away from the tubules in culture. After checking the digested tubules under a microscope to confirm the collagen layer had been permeabilized, the digested tubules were spun down to wash away the enzymes, and then resuspended in Sertoli cell media containing retinoic acid from ScienCell (4521) (ScienCell Research Laboratories, CA USA). The digested tubules were plated into six culture flasks in order to have three replicates of both control and treated cells for each exposure experiment. To remove contaminating germ cells from this primary culture, the cells were treated with hypotonic shock treatment on the morning of the second day of culture, and the enriched Sertoli cells were washed and allowed to recover with fresh media for at least two hours. The enriched primary cultures of Sertoli cells were then ready to be used for chemical exposure experiments. The estimated purity of the culture was >80% based on ICC staining for the Sertoli cell markers SOX9 and WT1 (Figure 2—figure supplement 2). A full procedure for the establishment of primary cultures of Sertoli cells can be found in Supplementary file 4.

#### Granulosa cell culture

Primary cultures of granulosa cells were established by selecting early preantral primary follicles from enzymatically digested ovary tissue as described previously (Monti and Redi, 2016; Roy and Greenwald, 1996), followed by breaking selected primary follicles down into a single cell suspension that could be plated and maintained. To isolate preantral primary follicles we dissected ovaries from ~5 female littermate mice at P20 and then enzymatically digested the ovary using collagenase IV (560 U/pair of ovaries) for 25 min at 37 °C with constant agitation. This digestion was stopped by addition of buffer with 0.5% BSA and the mixture was spun down at 60 x *g* for 5 min to pellet the cells. The cells were resuspended in PBS with 0.5% BSA and transferred to a sterile petri dish under a stereomicroscope. Primary follicles were individually picked from the solution using a glass needle with suction control and moved into a clean droplet of PBS containing 0.5% BSA. Selected primary follicles were then spun down at 1000 x *g* for 5 min. After spinning, the supernatant was removed, and the cells were finger-vortexed to resuspend the pellet. The follicles were then digested to single cell suspension by addition of pre-warmed (37 °C) 0.25% trypsin and incubated for 5 min with regular pipetting followed by pelleting again at 1000 x *g* for 5 min. The trypsin-containing supernatant was removed, and the granulosa cells were resuspended in DMEM supplemented with 15% FBS and plated into ix culture flasks in order to have three replicates of both control and treated cells for each exposure experiment. The oocytes were non-adherent and were washed away on day 2 when changing the media. The estimated purity of the culture was >90% based on ICC staining for the granulosa cell marker FSHR and INHA (Figure 2—figure supplement 2). The granulosa cells were then ready to be used for EDC exposure experiments. A full procedure for the establishment of primary cultures of granulosa cells can be found in Supplementary file 5.

#### Primordial germ cell like cell culture

Mouse iPSCs were differentiated into PGCLCs as previously described (Hayashi et al., 2011). Briefly, iPSCs maintained in N2B27 supplemented with 2i and LIF under feeder-free conditions were differentiated to EpiLCs for two days by the addition of activin A and basic fibroblast growth factor (bFGF) to the N2B27 media. After 2 days, the EpiLC intermediate cells were passaged to low adherence round bottom plates for 4 days in GK15 media containing bone morphogenic protein 4 (BMP4), stem cell factor (SCF), epidermal growth factor (EGF), and LIF to induce the differentiation of a subset of cells (2–3%) in the resulting aggregate to become PGCLCs. 6 batches each being made up of six plates were required in order to obtain sufficient cell numbers for three replicates of both control and treated conditions. During this 4-day period, cell aggregates were ready for EDC exposure (see below). Following EDC exposure, cell aggregates were removed from the low adherence round bottom plates within each batch, dissociated into a single cell suspension, and PGCLCs were fluorescence-activated cell sorted (FACS) on a BD FACSAria II in the UTSA Cell Analysis Core to recover cells that were double-positive for FUT4 and ITGB3. A full list of the media components and catalog numbers for the differentiation of iPSCs to PGCLCs along with our FACS gating for double positive FUT/ITGB3 PGCLCs can be found in Supplementary file 3 and Figure 2—figure supplement 3, respectively. Additional data demonstrating validation of PGCLCs by qRT-PCR and ICC staining are shown in Figure 7—figure supplement 3.

### Immunocytochemistry (ICC)

Cells were immunolabeled as previously described (Rodig, 2022) to validate the purity of cell primary cultures using known cell specific markers (Figure 2—figure supplement 2) to detect the presence or absence of relevant endocrine receptors ERα and ERβ at the protein level. Pluripotent (iPS) and somatic (Sertoli and granulosa) cells were grown on 13 mm Thermanox plastic coverslips (174950) prior to fixation, permeabilization, and immunolabeling (Nalge Nunc International, NY USA). Non-adherent germ (PGC and PGCLC) cells were spun down onto poly-L-lysine coated slides (63410–01) at 400 RPM for one minute using a Thermo Shandon CytoSpin III Cytocentrifuge from Rakin (Electron Microscopy Sciences, PA USA & Rakin Biomedical Corporation, MI USA). Cells were fixed with 4% formaldehyde for 10 min at room temperature (RT) and then washed three times for five minutes each with ICC buffer (PBS containing 0.01% Triton X-100 detergent) to permeabilize the cell and nuclear membranes prior to blocking with 5% goat serum which was added to the ICC buffer and incubated for 1 hr at RT. Following blocking, primary antibodies, in ICC buffer, were added to the slides and left to incubate overnight at 4 °C. The following day, slides were washed three times with ICC buffer, and fluorescent secondary antibodies in ICC buffer were then added to the slides and left to incubate in the dark for 1 hr. After secondary antibody incubation, cellular nuclei were stained with DAPI at a 1:1000 dilution in ICC buffer for 7 min, followed by three final washes with ICC buffer for 5 min. Coverslips then were transferred to microscope slides and mounted with 5–10 μL of VECTASHEILD Antifade Mounting Medium (H-1000) and sealed with clear nail polish before being imaged on a Zeiss AXIO Imager.M1 Fluorescence Microscope (Vector Laboratories Inc, CA, USA and Zeiss Group, Oberkochen DE). Images were processed for contrast and brightness enhancement and for the addition of scale bars using Fiji (RRID:SCR_002285; Schindelin et al., 2012). Information about the primary and secondary antibodies along with catalogue numbers and dilutions used can be found in the Key resources table.

### Quantitative RT-PCR (qRT-PCR)

Total RNA was extracted from 3 replicate preps of cells as described previously (Rio et al., 2010) and treated with 1.5 U/μg total RNA RQ1 DNase1 (M6101) to remove contaminating genomic DNA (Promega Corporation, WI USA). Fifty ng of cleaned RNA was retrotranscribed with SuperScript III as recommended by the manufacturer (Invitrogen, MA, USA). Primers were designed using Primer-BLAST (RRID:SCR_003095; Ye et al., 2012) from NCBI. A complete list of all primer sequences used in this study can be found in Supplementary file 6. Relative expression levels of selected genes were assessed by real-time PCR using the PowerTrack SYBR Green Master Mix according to the manufacturer’s instructions (Applied Biosystems, MA USA) then run on a QuantStudio 5 Real-Time PCR System (Thermo Fisher Scientific, MA, USA) and analyzed with QuantStudio Design and Analysis Software. Each sample was normalized based on constitutive expression of the *Gusb* reference gene to obtain the ΔCt [(2-Ctgene-CtGusb)].

### BPS exposure design

Despite numerous studies illustrating the dangers of estrogenic mimetic EDCs, the EPA has currently not published any limits concerning the maximum acceptable dose of BPS below which exposure on a daily basis is considered to be safe. Thus for this study, we established our initial testing range of 1 μM, 50 μM, and 100 μM of BPS based on the limit established for BPA at ≤ 4.44 μM (Ribeiro et al., 2019) selecting 1 dose below the established safe limit and two doses that exceed that limit. For all cell types except PGCLCs, BPS was dissolved in absolute ethanol and added to media gassed with 5% CO_2_, 5% O_2_, and balanced N_2_ and then injected into three replicate sealed T-25 cell culture flasks and left to incubate for 24 hr (Figure 1—figure supplement 3). The media was gassed to ensure enrichment to 5% CO_2_, which is normally regulated by the cell incubator, limiting the potential for the pH to change in the media during this exposure period due to the absorption of CO_2_ by incubating cells. The concentration of either BPS dissolved in ethanol for treatment groups or ethanol vehicle alone for control groups made up 0.02% of the total media volume. Following 24 hr of exposure, media containing BPS was removed and cells were washed and allowed to recover in fresh untreated media (without BPS or EtOH) for 24 hr prior to harvesting. PGCLCs were not suitable for this exposure paradigm as EpiLCs undergo differentiation to PGCLCs in cell aggregates formed in low-adherence round-bottom 96-well plates and cannot be maintained in T-25 sealed flasks. Therefore, diluted BPS was added to PGCLC media and added to cells in three replicate batches of round-bottom 96-well plates to incubate for 24 hr prior to a shortened wash and ‘chase’ period of 8 hr prior to cell sorting.

### Methylation beadchip analysis

A total of 1 μg of extracted genomic DNA from each of three replicate exposure experiments for each cell type was bisulfite-converted with the EZ DNA Methylation Kit (D5001) and modified according to the manufacturer’s recommendations (Zymo Research, CA USA and Illumina, CA USA). These samples were run on the Infinium Mouse Methylation BeadChip Array following the Illumina Infinium HD Methylation protocol. This beadchip array includes 297,415 cytosine positions within the mouse genome (CpG sites, non-CpG sites, and random SNPs). The methylation score for each CpG is represented as a β-value which is a ratio of the fluorescence intensity ranging between 0 (unmethylated) and 1 (methylated). Arrays were scanned by HiScan (Illumina, CA, USA). Quality control (Figure 2—figure supplement 4) and downstream data processing of the.idat files was using the Sensible Step-wise Analysis of DNA Methylation BeadChips (SeSAMe) Bioconductor package (Ding et al., 2023; Triche et al., 2013; Zhou et al., 2022; Zhou et al., 2018). DNA methylation levels of differentially methylated cytosines (DMCs) are determined using mixed linear models. This general supervised learning framework identifies CpG loci whose differential methylation is associated with known control vs. treated co-variates. CpG probes on the array were defined as having differential changes that met both p-value and FDR ≤ 0.05 significant thresholds between treatment and control samples for each cell type analyzed. Additionally, we followed up our DNA methylation analysis of individual dCpGs by identifying differentially methylated regions (DMRs). DMRs were created by grouping all CpGs measured on the array into clusters using Euclidean distance (Ding et al., 2023; Triche et al., 2013; Zhou et al., 2022; Zhou et al., 2018). The p-values from the differential methylation of individual CpGs within the resulting CpG clusters were aggregated, and clusters were then filtered selecting for regions that contained a p-value ≤ 0.05 (Ding et al., 2023; Triche et al., 2013; Zhou et al., 2022; Zhou et al., 2018).

### RNA-seq

Total RNA was extracted from 3 replicate preps of cells using Trizol as previously described (Rio et al., 2010). Contaminating genomic DNA was removed by RQ1 DNase (M6101) treatment according to the manufacturer’s instructions (Promega Corporation, WI USA). RNA concentration was determined using Qubit (Q32855) and RNA integrity (RIN) scores were determined using tape station (5067–5576) according to the manufacturer’s instructions (Thermo Fisher Scientific Inc, MA, USA and Agilent Technologies, Inc CA, USA). Strand-specific RNA-seq libraries were prepared with the NEBNext Ultra II Directional RNA Library Prep Kit for Illumina sequencing (E7760S) according to the manufacturer’s protocol (New England Biolabs, MA, USA). Briefly, this process consisted of poly(A) RNA selection, RNA fragmentation, and double stranded cDNA generation using random oligo(dT) priming followed by end repair to generate blunt ends, adaptor ligation, strand selection, and polymerase chain reaction amplification to generate the final library. Distinct index adaptors were used for multiplexing samples across multiple sequencing lanes. Sequencing was performed on an Illumina NovaSeq 6000 instrument yielding sequences of paired end 2x50 base pair runs. Demultiplexing was performed with the Illumina Bcl2fastq2 program (Illumina, CA, USA).

### RNA-seq expression analysis

The quality of the fastq reads was checked using FastQC (RRID:SCR_014583; Andrews et al., 2023; de Sena Brandine and Smith, 2019; Figure 7—figure supplement 4). Reads were aligned to the mm10 mouse reference genome using the Rsubreads package to produce read counts (RRID:SCR_016945; Liao et al., 2019). These were then used for differential gene expression analysis using the edgeR package (RRID:SCR_012802; Chen et al., 2016; McCarthy et al., 2012; Robinson et al., 2010). Briefly, gene counts were normalized using the trimmed mean of M-value normalization (TMM) method before determining counts per million (CPM) values (Robinson and Oshlack, 2010). For a gene to be classified as showing differential gene expression between BPS-treated and EtOH vehicle-only control samples, a threshold of both a Benjamini-Hochberg adjusted p-value ≤0.05 and a false discovery rate (FDR)≤0.05 had to be met.

### EM-Seq

Genomic DNA was extracted from three replicates of cells as previously described (Sambrook and Russell, 2006). Smaller fragmented DNA (≤10 kb) and contaminating RNA were removed by cleaning the genomic DNA on a genomic DNA clean and concentrator-10 column (D4011), according to the manufacturer’s instructions (Zymo Research, CA, USA). DNA concentration was determined using a Qubit (Q32850) and genomic DNA (100 ng) was sheered using a Bioruptor and the size of the sheered DNA was determined using a TapeStation 4200 according to the manufacturer’s instructions in the UTSA Genomics Core (Diagenode Inc, NJ, USA and Agilent Technologies, Inc CA, USA). Large size (470–520 bp) EM-seq libraries were prepared with the NEBNext Enzymatic Methyl-seq kit (E7120S) according to the manufacturer’s protocol (New England Biolabs, MA, USA). Briefly, this process consisted of A-tailing, adaptor ligation, DNA oxidation by TET2 initiated by the addition of Fe (II), strand denaturization with formamide, deamination by APOBEC3A, polymerase chain reaction amplification, and bead selection to generate the final libraries. Distinct index adaptors were used for multiplexing samples across multiple sequencing lanes. Sequencing was performed on an Illumina NovaSeq 6000 instrument yielding sequences of paired end 2x150 base pair runs. Demultiplexing was performed with the Illumina Bcl2fastq2 program (Illumina, CA, USA).

### EM-Seq analysis of genome-wide DNA methylation patterns

EM-seq data was processed using the comprehensive wg-blimp v10.0.0 software pipeline (Lehle and McCarrey, 2023; Wöste et al., 2020). In brief, reads were trimmed prior to initiating the wg-blimp pipeline using Trim Galore (RRID:SCR_011847; https://www.bioinformatics.babraham.ac.uk/projects/trim_galore/). Sequenced reads were aligned to the mm10 genome using gemBS (King et al., 2020; Merkel et al., 2019; Schilbert et al., 2020). The BAM files from alignment underwent a series of QC tests including read quality scoring by FastQC (RRID:SCR_014583; Andrews et al., 2023), overall and per-chromosome read coverage calculation, GC content, duplication rate, clipping profiles by Qualimap (RRID:SCR_001209; Okonechnikov et al., 2016), and deduplication by the Picard toolkit (RRID:SCR_006525; Figure 7—source data 1). Methylation calling was performed by MethylDackel (Ryan, 2023a; https://github.com/dpryan79/MethylDackel, copy archived at Ryan, 2023b) and statistically significant DMC/DMR calling was performed by the metilene (Jühling et al., 2016) and BSmooth (RRID:SCR_005693; Hansen et al., 2012) algorithms. Metilene uses a binary segmentation algorithm combined with a two-dimensional statistical test that allows the detection of DMCs/DMRs in large methylation experiments with multiple groups of samples. BSmooth uses a local-likelihood approach to estimate a sample-specific methylation profile, then computes estimates of the mean differences and standard errors for each CpG to form a statistic similar to that used in a t-test. Finally, potential regulatory regions were identified through the use of MethylSeekR (RRID:SCR006513; Burger et al., 2013). Results from the pipeline were displayed in the wg-blimp interactive results web browser that was built using the R Shiny local browser hosting framework (RRID:SCR_001626; Chang, 2023).

## Data Availability

Methylation beadchip, EM-seq, and RNA-seq data discussed in this publication have been uploaded and can be obtained from the SRA BioProject PRJNA1026145 and GEO accession GSE252723. The following datasets were generated: LehleJD LinY-H GomezA ChavezL McCarreyJR 2024DNA methylation dataNCBI BioProjectPRJNA1026145 LehleJD LinY-H GomezA ChavezL McCarreyJR 2024Gene expression dataNCBI Gene Expression OmnibusGSE252723

## Additional files

MDAR checklist

Supplementary file 1. Persisting DEGs in PGCLCs derived from iPSCs exposed to 1uM BPS.

Supplementary file 2. Validation of normal karyotype analysis of MF5-9-1 iPSCs. iPSCs from reprogrammed MEFs were validated for a normal karyotype prior to use in this project.

Supplementary file 3. iPSC, EpiLC, and PGCLC Culture Media Components.

Supplementary file 4. Preparation of primary cultures of Sertoli cells from mice.

Supplementary file 5. Preparation of primary cultures of granulosa cells from mice protocol.

Supplementary file 6. qRT-PCR primers.

## Appendix 1

**Appendix 1—key resources table:**

Reagent type (species) or resource	Designation	Source or reference	Identifiers	Additional information
Genetic reagent (*M. musculus*)	R26^rtTA^; Col1a1^2lox-4F2A^	The Jackson Laboratory	011011	
Cell line (*M. musculus*)	CF1 Mouse embryonic fibroblasts, MitC-treated	Thermo Fisher Scientific	A34959	
Chemical compound	Dulbecco’s Modified Eagle Medium (DMEM)	Thermo Fisher Scientific	10313021	High glucose, pyruvate, no glutamine
Chemical compound	Fetal bovine serum (FBS)	Thermo Fisher Scientific	10439024	Embryonic stem-cell FBS, qualified, USDA-approved regions
Chemical compound	Leukemia inhibitory factor (LIF)	Millipore Sigma	ESG1107	ESGRO Recombinant Mouse LIF Protein
Chemical compound	DMEM/F12	Thermo Fisher Scientific	21041025	No phenol red
Chemical compound	Insulin	Millipore Sigma	I1882	From bovine pancreas
Chemical compound	Apo-Transferrin	Millipore Sigma	T1147	From human
Chemical compound	Bovine Serum Albumin (BSA)	Thermo Fisher Scientific	15260037	Fraction V (7.5% solution)
Chemical compound	Progesterone	Millipore Sigma	P8783	
Chemical compound	Putrescine dihydrochloride	Millipore Sigma	P5780	
Chemical compound	Sodium selenite	Millipore Sigma	S5261	
Chemical compound	Neurobasal	Thermo Fisher Scientific	12348017	No phenol red
Chemical compound	B-27	Thermo Fisher Scientific	12587010	(50 X), minus vitamin A
Chemical compound	Penicillin-streptomycin	Thermo Fisher Scientific	15070063	(5,000 U/mL)
Chemical compound	GlutaMAX	Thermo Fisher Scientific	35050061	(100 X)
Chemical compound	2-Mercaptoethanol	Thermo Fisher Scientific	21985023	(1000 X)
Chemical compound	CHIR99021	BioVision	1677–5	
Chemical compound	PD0325901	Amsbio	04-0006-02	10 mM in DMSO
Chemical compound	Recombinant Human/ Murine/Rat Activin A	PeproTech	120–14	Insect derived
Chemical compound	Recombinant Human FGF-Basic (FGF-2/bFGF)	Thermo Fisher Scientific	13256–029	
Chemical compound	KnockOut Serum	Thermo Fisher Scientific	10828028	
Chemical compound	Glasgow's MEM (GMEM)	Thermo Fisher Scientific	11710035	
Chemical compound	Recombinant human bone morphogenetic protein 4 (BMP-4)	R&D Systems	314 BP-010	
Chemical compound	Recombinant mouse stem cell factor (SCF)	R&D Systems	455-MC-010	
Chemical compound	Recombinant human epidermal growth factor (EGF), carrier free (CF)	R&D Systems	2028-EG-200	
Chemical compound	Dulbecco’s phosphate- buffered saline (DPBS)	Thermo Fisher Scientific	14040133	
Chemical compound	Deoxyribonuclease I (DNaseI)	Millipore Sigma	DN25	From bovine pancreas
Chemical compound	Trypsin (2.5%)	Thermo Fisher Scientific	15090046	No phenol red
Chemical compound	Soybean trypsin inhibitor	Thermo Fisher Scientific	17075029	
Chemical compound	Collagenase type IV	Worthington	LS004188	From Clostridium histolyticum
Chemical compound	Sertoli Cell Medium	ScienCell Research Laboratories	4521	
Chemical compound	Ethanol (EtOH)	Fisher	BP28184	(200 Proof)
Chemical compound	BSA	Millipore Sigma	A9085	
Chemical compound	Heat inactivated (HI) FBS	Thermo Fisher Scientific	10082147	
Chemical compound	Trypsin-EDTA (0.25%)	Thermo Fisher Scientific	25200072	With phenol red
Chemical compound	Bisphenol S (BPS)	Millipore Sigma	43034–100 MG	
Chemical compound	Phenol:Chloroform:Isoamyl Alcohol (25:24:1, v/v)	Thermo Fisher Scientific	15593031	
Chemical compound	TRIzol	Thermo Fisher Scientific	15596026	
Chemical compound	Isopropanol	Thermo Fisher Scientific	327272500	
Chemical compound	Proteinase K Solution (20 mg/mL)	Thermo Fisher Scientific	25530049	
Chemical compound	MaXtract High Density	Quiagen	129046	Phase lock gel tubes
Chemical compound	Sodium Acetate Solution	Thermo Fisher Scientific	R1181	3 M, pH 5.2
Chemical compound	Glycogen (5 mg/ml)	Thermo Fisher Scientific	AM9510	
Chemical compound	NaCl	Thermo Fisher Scientific	J21618.36	
Chemical compound	Tris base	Millipore Sigma	77-86-1	
Chemical compound	Ethylenediaminetetraacetic acid (EDTA)	Millipore Sigma	E9884-100G	
Chemical compound	Sodium dodecyl sulfate (SDS)	Millipore Sigma	151-21-3	
Chemical compound	Triton X-100	Thermo Fisher Scientific	85111	
Chemical compound	RQ1 DNase	Promega	M6101	
Chemical compound	Propidium iodide	BioLegend	421301	FCy 5 μL/10^6^ cells
Antibody	ERα	Thermo Fisher Scientific	MA1-310	Host: mouse monoclonal, ICC 1:100
Antibody	ERβ	GeneTex	GTX70174	Host: mouse monoclonal, ICC 1:100
Antibody	INHA	Invitrogen	PA5-13681	Host: rabbit polyclonal, ICC 1:25
Antibody	FSHR	Affinity	AF5477	Host: rabbit polyclonal, ICC 1:250
Antibody	SOX9	Abcam	ab185966	Host: rabbit monoclonal, ICC 1:100
Antibody	GAPDH	Novus	NB300-221	Host: mouse monoclonal, ICC 1:100
Antibody	WT1	Novus	NBP2-67587	Host: rabbit monoclonal, ICC 1:100
Antibody	FUT4	GeneTex	GTX34467	Host: rabbit monoclonal, ICC 1:50
Antibody	NANOG	Abcam	ab80892	Host: rabbit polyclonal, ICC 1:100
Antibody	POU5F1	Abcam	ab19857	Host: rabbit polyclonal, ICC 1:200
Antibody	SOX2	Abcam	ab97959	Host: rabbit polyclonal, ICC 1:200
Antibody	ID4	Thermo Fisher Scientific	PA5-26976	Host: rabbit polyclonal, ICC 1:50
Antibody	AR	Santa Cruz	sc-7305	Host: mouse monoclonal, ICC 1:50
Antibody	PPARγ	Santa Cruz	sc-7273	Host: mouse monoclonal, ICC 1:200
Antibody	RXRα	Invitrogen	433900	Host: mouse monoclonal, ICC 1:200
Antibody	PRDM1	Thermo Fisher Scientific	14-5963-82	Host: rat monoclonal, ICC 1:50
Antibody	Goat Anti Mouse Alexa 647	Abcam	ab150119	Host: goat polyclonal, ICC 1:200
Antibody	Goat Anti Rabbit Alexa 488	Abcam	ab150081	Host: goat polyclonal, ICC 1:1000
Antibody	Goat Anti Rabbit Alexa 647	Abcam	ab150179	Host: goat polyclonal, ICC­­ 1:200
Antibody	Goat Anti Rat Alexa 647	Thermo Fisher Scientific	A21247	Host: goat polyclonal, ICC 1:200
Antibody	FUT4 (IgM, κ), brilliant violet 421	BD Horizon	562705	Host: mouse monoclonal, FCy 5 μL/10^6^ cells
Antibody	ITGB3 (IgG), PE	BioLegend	104307	Host: hamster monoclonal, FCy 1 μL/10^6^ cells
Antibody	IgM, κ Isotype control, brilliant violet 421	BD Horizon	562704	Host: mouse monoclonal, FCy 1.25 μL/10^6^ cells
Antibody	IgG Isotype control, PE	BioLegend	400907	Host: hamster monoclonal, FCy 1 μL/10^6^ cells
Commercial assay or kit	RNA Clean & Concentrator-5	Zymo Research	R1016	
Commercial assay or kit	Genomic DNA Clean & Concentrator-10	Zymo Research	D4011	
Commercial assay or kit	EZ DNA Methylation Kit	Zymo Research	D5001	
Commercial assay or kit	SuperScript III One-Step RT-PCR System with Platinum Taq DNA Polymerase	Thermo Fisher Scientific	12574026	
Commercial assay or kit	PowerTrack SYBR Green Master Mix for qPCR	Thermo Fisher Scientific	A46109	
Commercial assay or kit	Infinium Mouse Methylation BeadChip	Illumina	20041558	
Commercial assay or kit	RNA ScreenTape & Reagents	Agilent	5067–5576	
Commercial assay or kit	DNA ScreenTape & Reagents	Agilent	5067–5583	
Commercial assay or kit	Qubit dsDNA (Broad Range) BR Assay Kit	Thermo Fisher Scientific	Q32850	
Commercial assay or kit	Qubit RNA (high sensitivity) HS Assay Kit	Thermo Fisher Scientific	Q32855	
Commercial assay or kit	NEBNext Ultra II Directional RNA Library Prep Kit for Illumina	New England BioLabs	E7765	
Commercial assay or kit	NEBNext Poly(A) mRNA Magnetic Isolation Module	New England BioLabs	E3370	
Software, algorithm	ZEISS ZEN Microscopy Software	https://www.zeiss.com/microscopy/en/products/software/zeiss-zen.html	ZEN 3.7	RRID:SCR_013672
Software, algorithm	Primer-BLAST	https://www.ncbi.nlm.nih.gov/tools/primer-blast/		RRID:SCR_003095
Software, algorithm	QuantSudtio Design & Analysis Software	https://www.thermofisher.com/us/en/home/technical-resources/software-downloads/quantstudio-3-5-real-time-pcr-systems.html	QuantStudio v1.5.1	
Software, algorithm	Fiji	https://fiji.sc/	Fiji v1.54f	RRID:SCR_002285; Schindelin et al., 2012
Software, algorithm	Bfastq2	https://support.illumina.com/downloads/bcl2fastq-conversion-software-v2-20.html	Bcl2fastq2 v2.20	
Software, algorithm	FastQC	https://www.bioinformatics.babraham.ac.uk/projects/fastqc/	FastQC 0.12.0	RRID:SCR_014583; Andrews et al., 2023; Smith and de Sena Brandine, 2021
Software, algorithm	Wg-blimp	https://github.com/MarWoes/wg-blimp	Wg-blimp v0.10.0	Lehle and McCarrey, 2023; Wöste et al., 2020
Software, algorithm	R-Project for Statistical Computing	http://www.r-project.org/	R 4.2.1	Packages: SeSAMe Ding et al., 2023; Triche et al., 2013; Zhou et al., 2022, Zhou et al., 2018, stringr RRID:SCR_022813; Wickham and RStudio, 2022, kintr RRID:SCR_018533; Xie et al., 2023, SummarizedExperiment Morgan et al., 2023, ggrepel RRID:SCR_017393; Slowikowski et al., 2023, pals Wright, 2023, wheatmap Zhou, 2022, magrittr Bache et al., 2022, ggplot2 RRID:SCR_014601; Wickham et al., 2023a, dplyr RRID:SCR_016708; Wickham et al., 2023b, tidyr RRID:SCR_017102; Wickham et al., 2023c ggvenn RRID:SCR_025300; Yan, 2023, RColorBrewer RRID:SCR_016697; Neuwirth, 2022, RIdeogram Hao et al., 2020, AnnotationDbi RRID:SCR_023487; Pagès et al., 2023, Mus.musculus Team, 2015, BSgenome.Mmusculus.UCSC.mm10 Team, 2021, GenomicRanges RRID:SCR_000025; Lawrence et al., 2013, universalmotif Tremblay, 2023, memes RRID:SCR_001783; Nystrom, 2023, plyranges RRID:SCR_021324; Lee et al., 2019, rtracklayer RRID:SCR_021325; Lawrence et al., 2009, Rsubread RRID:SCR_016945; Liao et al., 2019, edgeR RRID:SCR_012802; Chen et al., 2016; McCarthy et al., 2012; Robinson et al., 2010
