# Supplementary material for: An in vitro approach reveals molecular mechanisms underlying endocrine disruptor-induced epimutagenesis
Source: eLife. 2024 Oct 3;13:RP93975. doi: 10.7554/eLife.93975 (PMC11449486; doi:10.7554/eLife.93975)
Supplement: Supplementary file 2. — iPSCs from reprogrammed MEFs were validated for a normal karyotype prior to use in this project. [file elife-93975-supp2.docx]

**Supplementary file 2.**

**
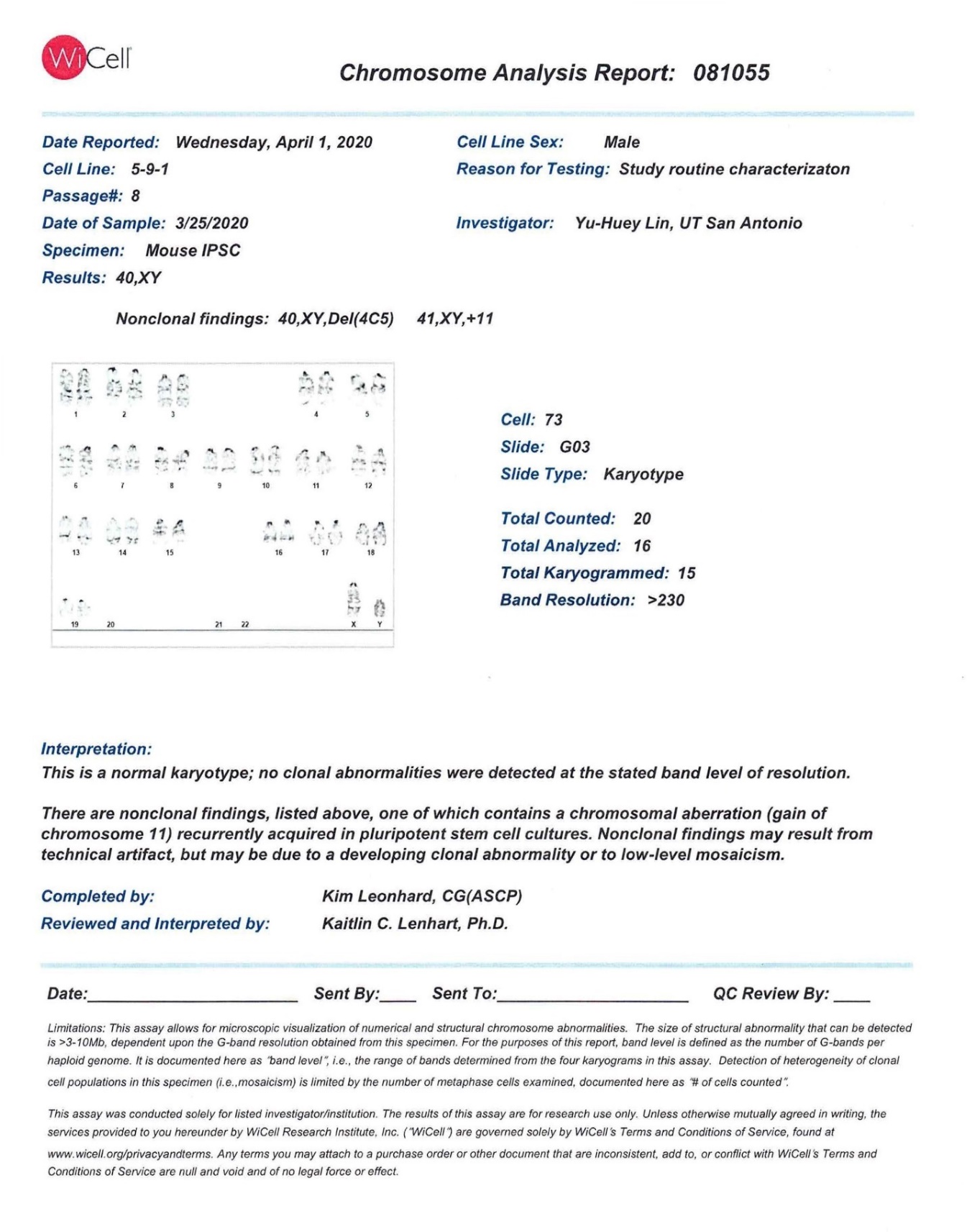
**

**Validation of normal karyotype analysis of MF5-9-1 iPSCs.** iPSCs from reprogrammed MEFs were validated for a normal karyotype prior to use in this project.
